# Structure Elucidation and Botanical Characterization of Diterpenes from a Specific Type of Bee Glue

**DOI:** 10.3390/molecules22071185

**Published:** 2017-07-14

**Authors:** Noushin Aminimoghadamfarouj, Alireza Nematollahi

**Affiliations:** Faculty of Pharmacy A15, The University of Sydney, Sydney, NSW 2006, Australia; nami1357@uni.sydney.edu.au

**Keywords:** *Myoporum insulare*, Scrophulariaceae, serrulatanes, bee glue, diterpenes

## Abstract

Investigation of the single plant source bee glue type originating from Southern Australia resulted in the isolation and structure elucidation of major serrulatane diterpenes, novel 7,8,18-trihydroxyserrulat-14-ene (**1**), along with its oxidized product, 5,18-epoxyserrulat-14-en-7,8-dione (**3**) and known (18*RS*)-5,18-epoxyserrulat-14-en-8,18-diol (**2**). Exploration into the botanical origin revealed *Myoporum insulare* R. Br, as the plant source of the bee glue materials. This discovery was made through comparative analysis of the myoporum bee glue samples collected from the beehives, analyses of plant resinous exudate, and resin carried on the hind legs of bees foraging for bee glue.

## 1. Introduction

Bee glue (Propolis) is a resinous substance collected by worker honey bees, *Apis mellifera*, from specific plants and transported to the hives where it is mixed by bees with beeswax to form strongly adhesive material with antiseptic properties to aid in defense and for maintenance of hygiene in the hive [1,2,3]. There are well-documented instances of its usage in history i.e., Egyptians have used propolis to mummify corpses based on its preservation properties. Abu Ali Sina (Avicenna) the Persian scientist has mentioned propolis as the black wax [4]. Similarly, in other parts of the world such as South Africa, Eastern Europe, France, and Russia traditional folk medicine use of it has been recognized [5,6,7,8]. The chemical composition of the propolis depends on its plant source which varies with geographical location. There is no information showing that bees do any significant chemical processing on the collected resins [9]. The chemical groups of compounds identified in propolis samples include: terpenes, lignans, flavonoids, chalcones, stilbenes, cinnamates, aromatic acids and esters, aliphatic acids and esters, benzophenones, benzofurans and sugars [10,11]. Due to its unique flora, the Southern part of Australia (Kangaroo Island) has been recognized as an area rich in bees’ propolis [12]; therefore, a phytochemical analysis was done to categorize propolis types collected from different regions of the island. Two serrulatane diterpenes along with one oxidized product were isolated from a specific propolis type designated as “purple spot” and later called myoporum propolis after identification of its botanical source as *Myoporum insulare* R. Br. from the plant family of Scrophulariaceae Juss. Chemical structure elucidation was determined from 1D/2D NMR and ^1^H-NMR decoupling experiments, aided by comparison with published X-ray crystallographic data of serrulatane diterpenes [13,14,15]. The confirmation of the myoporum propolis samples’ botanical origin was completed by the determination of their chemical components along with observation of bees’ behavior and by analysis of the island flora. 

## 2. Results

### 2.1. Structure Elucidation of Serrulatanes from Myoporum Bee Glue

**Figure 1:**
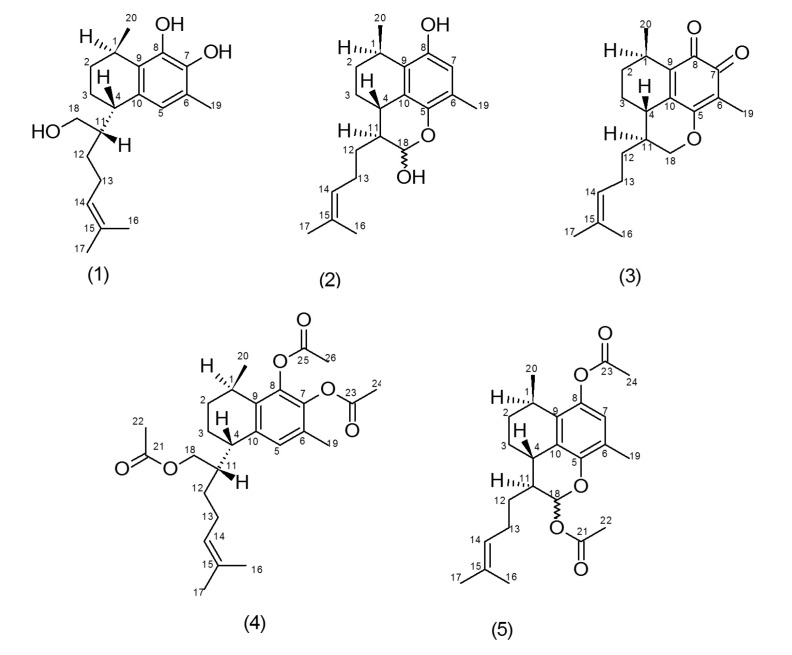
Chemical structures of the diterpenes, compounds **1–5** from the myoporum bee glue.

Propolis samples were collected broadly over the Island with beekeeping practice modified to minimize mixing of propolis from different plant sources. Propolis samples from different apiary sites were never mixed and collection from each site was done over a relatively short period of time i.e., approximately two weeks. To further minimize mixing from different plant sources, propolis collections were restricted to a maximum of 1 kg per sample bag and every bag was analyzed. More than 1000 samples were analyzed by thin-layer chromatography (TLC) and ^1^H-NMR studies and classified into six main propolis types; three types have been published [16,17] and the others are yet to be published [18]. Multiple propolis samples based on their relatively simple TLC and NMR pattern were identified as a likely single-source propolis of a type and initially named “purple spot” propolis (myoporum propolis) based on a characteristic purple spot observed by TLC and also purple coloration of the propolis and its ethanol solutions. This propolis type was extracted and fractionated by normal-phase short-column vacuum chromatography (NP-SCVC) to give two major components identified as novel **1** and known **2** diterpenes (Figure 1) from the interpretation of 1D/2D NMR spectroscopic and high-resolution electrospray ionization mass spectrometry (HRESIMS) data.

Compound **1** was a yellow liquid identified as 7,8,18-trihydroxyserrulat-14-ene. Its molecular mass was recorded as *m*/*z* 341.2089 [M + Na]^+^ (calcd. 341.2087) by HRESIMS analysis, hence the molecular formula was concluded as C_20_H_30_O_3_. With the use of ^1^H-NMR, ^13^C-NMR, HSQC, HMBC and ^1^H-^1^H COSY spectra, the chemical structure **1** was determined (Table 1 and Table 2).

**Figure 2:**
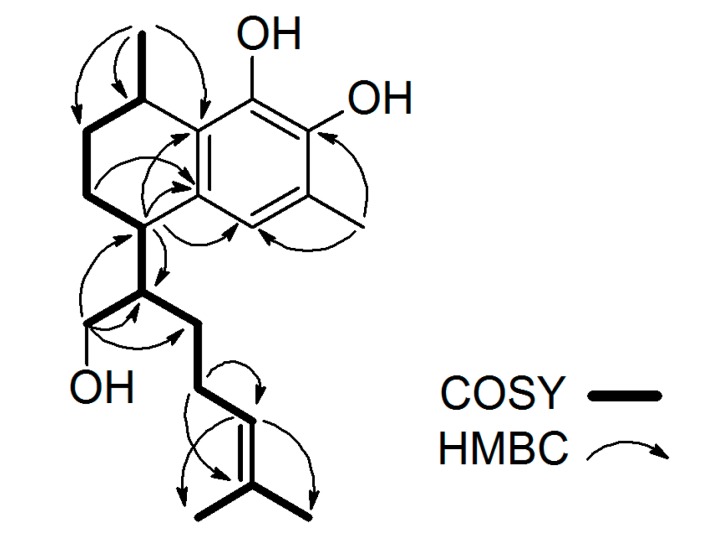
^1^H-^1^H COSY and HMBC correlations for compound **1**.

Analysis of ^1^H-^1^H COSY spectrum indicated the backbone relations from methyl group at position 20 to the di-methylated (C-16 and C-17) olefin moiety demonstrated by the bold lines. HMBC spectrum also confirmed the observed correlations (Figure 2). From the HMBC data strong correlation was noted for the methyl group at H-20 (δ_H_ 1.21) with C-1 (δ_c_ 26.9). Additionally, strong HMBC correlations were detected for δ_H_ 5.04 (H-14) to C-16 (δ_c_ 25.7) and C-17 (δ_c_ 17.7), which confirmed the position of the terminal di-methylated olefin moiety side chain. A one-proton singlet in the aromatic region at 6.56 ppm due to H-5 was noted in the ^1^H-NMR spectrum of compound **1**. At 5.04 ppm, a broad triplet split integrating for one proton was recorded with a *J* value of 7.0 Hz which matched with (H-14) of the isoprene sidechain part of the serrulatane diterpene structure. At 5.42 and 5.23 ppm, two broad one-proton singlets were assigned to the catechol hydroxyl moieties situated on the aromatic ring (OH-8, OH-7), respectively. A two-proton doublet at 3.63 ppm with *J* value of 6.16 Hz was assigned to position H_2_-18. The hydrogen at position 1 (H-1) showed a one-proton signal at 3.07 ppm with a splitting pattern of a quintuplet of doublets and *J* values of *J* = 6.8, 1.6 Hz. Furthermore, H-4 resonated as a one-proton triplet of doublets at 2.76 ppm (*J* values 5.6 and 2.6 Hz) plus the three hydrogens at chemical shift of 2.20 ppm were assigned to H-19 corresponding to the methyl group on the aromatic ring. The other two methyl groups were noted at 1.68 and 1.58 ppm, matching to H-16 and H-17, respectively.

Similar procedures as for compound **1** were followed to elucidate the structures of other isolated chemical compounds. Compound **2** was defined as (18*RS*)-5,18-epoxyserrulat-14-en-8,18-diol, with its HRESIMS showing molecular mass of *m*/*z* 315.1970 [M − H]^−^ (calcd. 315.1966) and a molecular formula of C_20_H_28_O_3_. 

Structures **1** and **2** present a high degree of similarity in terms of their ^1^H-NMR and ^13^C-NMR chemical shift values for their non-aromatic ring and the olefinic side chain (Table 1 and Table 2). One significant difference is the presence of C-18 methine group in **2** when compared to compound **1**, and the down-field shift of proton and carbon (δ_H_ 5.59 and δ_c_ 91.9) indicated the presence of C-18 hemiacetal methine group in **2**. One more change between **1** and **2** takes place at aromatic proton position, C-7 (δ_c_ 115.9) in **2** compared with C-5 (δ_c_ 122.0) in **1**.

Compound **3**, the oxidized product of **1**, with molecular mass of *m*/*z* 315.19573 [M + H]^+^ (calcd. 315.19547) based on HRESIMS, was assigned the molecular formula C_20_H_26_O_3_, and was identified as 5,18-epoxyserrulat-14-en-7,8-dione (Figure 1). It is proposed that compound **3** may be formed through a four-stage process with two stages of oxidation (Supporting information). The catechol moiety of **1** may be oxidized to an intermediate ortho-benzoquinone. In the second stage, the 18-hydroxyl group adds to the 5,6 double-bond to form the six-membered ring with the O-18 to C-5 bond. As a result of the addition, the intermediate has a 7,6-double-bond and a 7-hydroxy group. In the third stage, by keto to enol tautomersim, H-5 may be lost and a hydrogen gained on O-8 to give a catechol hydroxyl. The second catechol oxidation in the last stage gives compound **3**. On account of oxidation reaction, catechol hydroxyl hydrogens are lost in the formation of the benzoquinone keto groups at position 7 and 8, which make C-7 and C-8 less shielded with δ_c_ 179.4 and δ_c_ 181.9, respectively. Compound **3** lacks the aromatic proton at position 5 compared to compound **1** and also there are changes to the splitting pattern for protons in position 18. In more detail, H-18_a_ was detected at 3.86 ppm with the splitting pattern of a triplet and *J* value of 10.8 Hz and the other, H-18_b_, was noted at 4.49 ppm with *dd* splitting and *J* values of 10.8 and 3.6 Hz (Table 1). This pattern is consistent with the additional ring formed by oxidative cyclisation between position 5 and oxygen 18 to form an additional six-membered ring where H-18_a_ is *pseudoaxial* and H-18_b_ is *pseudoequatorial*.

### 2.2. Derivatization of Isolated Serrulatanes

To avoid quinonoid products formation of compound **1** and possible equilibrium of open and closed forms of compound **2** [19], for long duration NMR studies; rotating frame overhause effect spectroscopy (2D ROESY) and ^1^H-NMR decoupling, the hydroxyl groups were acetylated to provide respectively compounds **4** and **5** (Figure 1). The expanded and more specific C-NMR data from serrulatane derivatives enable a more definitive interpretation of the relative stereochemistry of the serrulatane core through closer comparison with the few available X-ray studies on serrulatane skeleton stereochemistry [13,14,15].

The acetylated compound **4**, isolated as a pale yellow oil, was determined to have a molecular mass of *m*/*z* 467.2405 [M + Na]^+^ (calcd. 467.2404) in the HRESIMS and a molecular formula established as C_26_H_36_O_6_. The experimental HMBC connection between H-11 (δ_H_ 2.1) and C-4 (δ_c_ 36.9) showed that the C-11 (side chain) is directly attached to the non-aromatic ring, this was also supported by a substantial ion at *m*/*z* 275.06 in the mass spectrum caused by the cleavage of C-4/C-11 which is frequently reported among serrulatane compounds [20]. HMBC correlations from δ_H_ 1.15 (H-20) to C-1 (δ_c_ 27.6) and C-9 (δ_c_ 134.1) suggested the attachment of the methyl group to the C-1 of the bicyclic system and this was further established by decoupling experiments. The H-4 neighboring to an aromatic ring was specified by HMBC correlations from δ_H_ 2.88 (H-4) to C-5 (δ_c_ 128.1), C-9 (δ_c_ 134.1) and C-10 (δ_c_ 137.1). The cyclohexene ring foundation was complete by showing H-1 (δ_H_ 2.92) HMBC correlations to C-9 and C-10. The strong HMBC correlation from δ_H_ 4.11 proton to a carbonyl moiety confirmed the attachment of this acetoxy group to this methylene moiety, and the distinct HMBC correlations to C-4, C-11 and C-12, positioned it at H-18. High level of uniformity was achieved when comparisons were made among the ^1^H and ^13^C-NMR data (Table 1 and Table 2) of the aromatic ring plus the side-chain assignment, with other similar previous literature data [13,21].

Searching similar substructures accompanied by consideration of diterpene biosynthetic pathways indicated that compound **4** possessed a serrulatane skeleton. Allocating the relative configuration of the serrulatanes using ^1^H-NMR data only is very challenging [13]. The relative configurations for the chiral centers (C-1, C-4 and C-11) were clarified on the basis of ^1^H-NMR decoupling and ROESY data. When irradiation of the H-1 proton was examined, the doublet (*J* = 6.9 Hz) protons of the methyl group at δ_H_ 1.15 collapsed to a broad singlet. The similarity of H-1 splitting pattern with reported serrulatanes [22] suggested a distorted chair conformation for the cyclohexene ring with H-1 in *pseudoequatorial* orientation. According to the ROESY correlations of H-1 and H-2_a_, these protons are located on the same face of the molecule. The *pseudoequatorial* configuration of H-4 was understood from the intensive ROESY correlation between H-4 and H-5, consistent with the reported X-ray structure (CCDC 999272) [14]. Subsequently, ROESY correlations between H-1/H-2_a_ and H-4/H-3_a_ positioned H-1 and H-4 on the opposed faces of the molecule. In relation to the negative specific rotation for the published serrulatanes in literature [15,22], 1R*, 4S* configuration was assigned, consistent with obtained optical rotation results for the isolated serrulatanes presented in this study. The flexibility of the isoprene side chain complicated the configuration determination at C-11, however, from the spatial ROESY correlation between H-4/H-11, the configuration of H-11 was established as 11*S*. Therefore, the concluding relative configuration of the compound **4** best fits as 1R*, 4S*, 11S*, in agreement with reported similar configurations [14,22,23,24,25,26,27,28] for the serrulatane skeleton.

After isolating compound **2**, elucidation difficulties were faced due to the hemiacetal cyclization and equilibrium of open and closed forms, therefore the acetylation of compound **2** was carried out, resulting in compound **5** with a molecular formula of C_24_H_32_O_5_ and molecular mass *m*/*z* 423.2143 [M + Na]^+^ (calcd. 423.2142) in the HRESIMS. Compound **5** ^1^H-NMR spectrum presented a similar pattern to **4**, with a significant doublet signal (*J* = 2.1 Hz) at δ_H_ 6.53 corresponding to the proton H-18 shifted to the lower field on forming the acetate, owing to the electron-withdrawing influence and anisotropy of the acetoxy group attached to position 18. ^1^H and ^13^C-NMR data (Table 1 and Table 2) were consistent with compound **5** being a serrulatane diterpene. The ^1^H and ^13^C-NMR spectra of compound **5** were almost identical with that of the published serrulatane [19], with the exception that in the side chain, the bond between C-14/C-15 was unsaturated. The relative configuration of compound **5** was determined as 1R*, 4S* and 11S* from the observed similarity with reported serrulatanes and X-ray crystal studies [13,14,15].

Molecular geometry affects ^13^C chemical shifts; hence, a possible underlying explanation for the downfield C-2 and C-3 chemical shifts noticed for structures **2**, **3** and **5** could be addressed as the steric effects caused by cyclization and rigidity among these three mentioned structures; whereas in case of compound **1** and its derivative (**4**), ^13^C chemical shifts at these positions are noted more upfield, same pattern has been reported for similar serrulatane diterpenes as published in literature [22,25].

### 2.3. Identification of Botanical Origin

**Figure 3:**
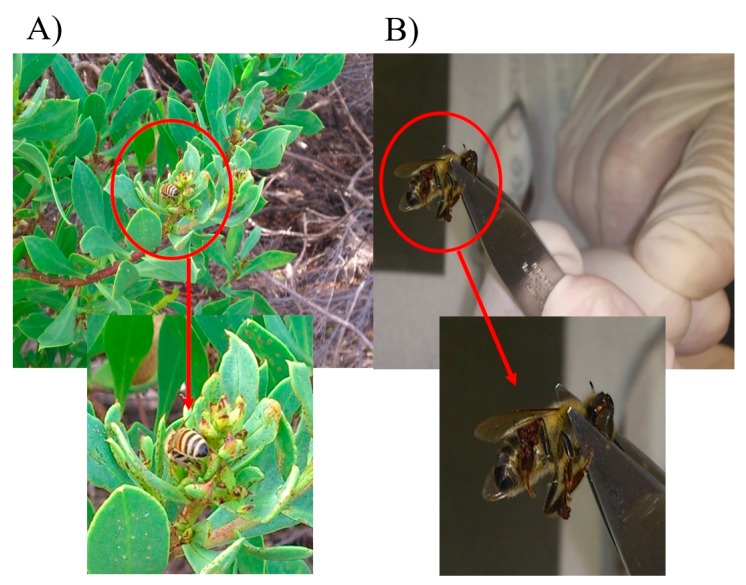
(**A**) Propolis collection by bees from resinous leaves of *Myoporum insulare*; (**B**) Laboratory analysis of the bee legs with propolis collected from *Myoporum insulare* leaves surface.

Focusing on the isolated chemical structures **1** and **2**, our research group concentrated on searching to find the botanical origin of the myoporum propolis type. From the main skeleton of the compounds, serrulatane, plants of the genus, *Eremophila*, Scrophulariaceae family, were considered as possible sources. Since *Eremophila* species are rare on the Island, plants of the closely related genus *Myoporum*, of widespread occurrence on the Island, were also taken into consideration. By means of the flora distribution map of the Island for these categories [29,30,31,32], and having the location of apiary sites in mind, the best regions were recognized. Subsequently, bees were detected collecting resin from the sticky buds and leaves to produce propolis from a shrub or tree, called *Myoporum insulare*, near coastal zones (Figure 3, Appendix B video supplementary data).

**Figure 4:**
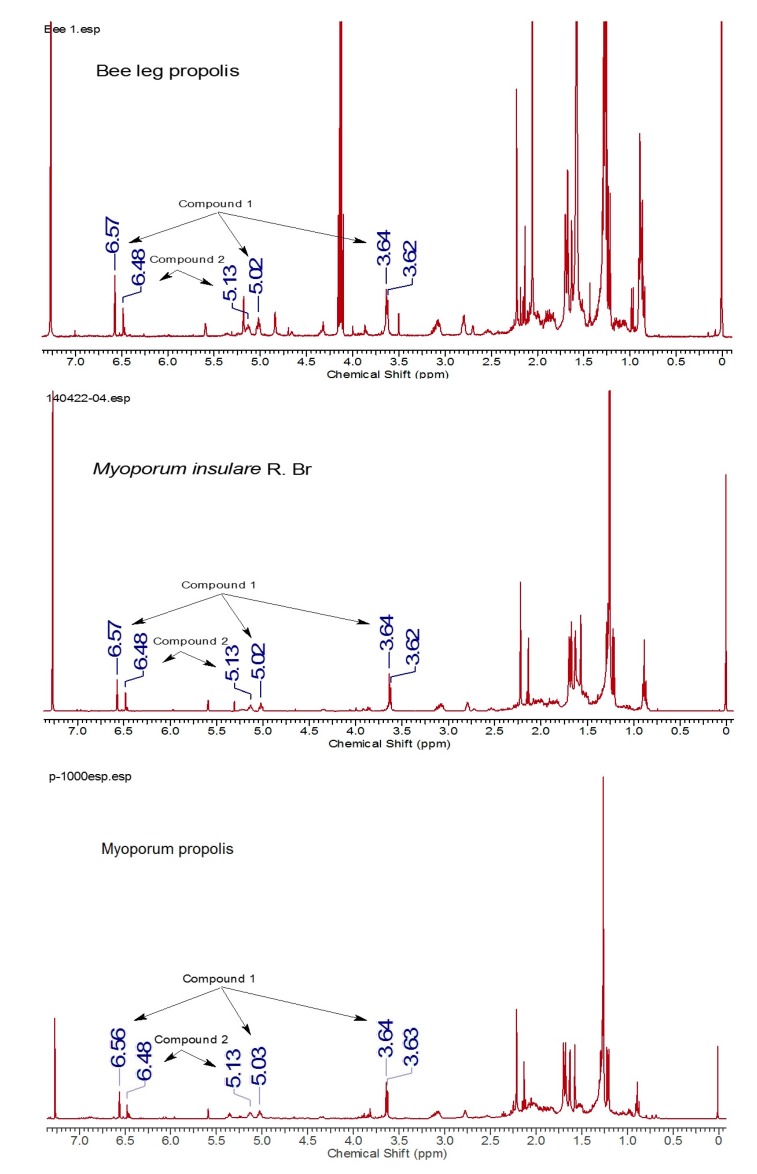
^1^H-NMR spectra for bee leg propolis, plant resin and myoporum propolis.

Plant samples were found in the vicinity of the beehives across the Island in sites where the myoporum propolis samples had been collected. Comparative NMR analysis of propolis on bee legs, myoporum propolis collected from the hives mats, plus the resin extracted from the shiny sticky sprout tips of the leaves of *Myoporum insulare* revealed identical chemical constituents’ profiles in comparable proportions (Figure 4). 

**Figure 5:**
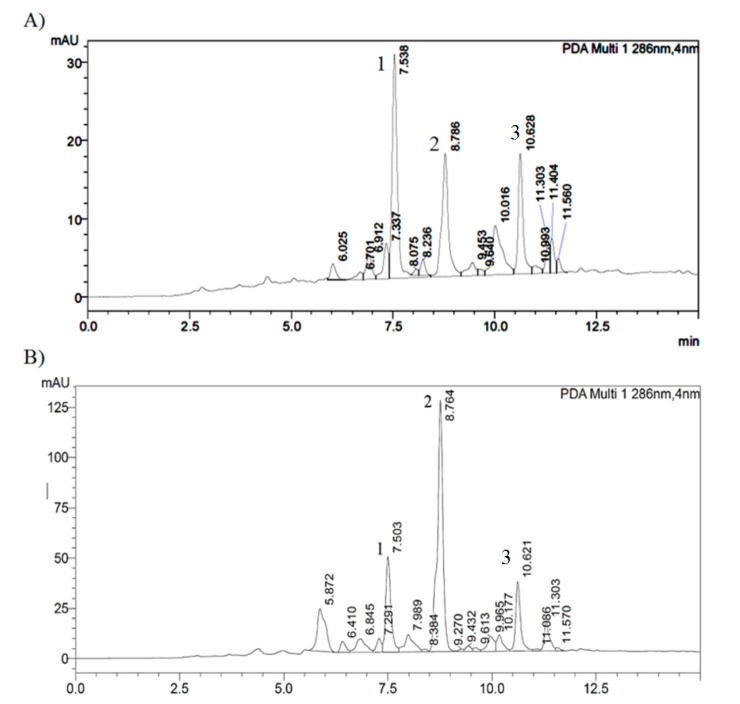
C-18 Reversed-phase HPLC chromatograms of (**A**) *Myoporum insulare* resinous leaf extract and (**B**) bee leg propolis extract. Compounds **1**–**3** are labeled. The gradient HPLC analysis was carried out using Shimadzu UFLC with a Hewlett Packard Column, NUCLEOSIL 100C18, 5 μm, 4 mm × 125 mm, eluted with mobile phase, consisted of methanol (phase A), and methanol: water: acetic acid (40:59.8:0.2) (phase B), detected at 286 nm with a UV-Vis detector Shimadzu SPD-20A.

Moreover, Figure 5 presents comparative HPLC profiling of the sticky leaf surface resin extract of *Myoporum insulare* and bee legs propolis extract.

## 3. Discussion

Considering the unique flora in Australia, only a small number of studies have been done on the Australian bee glue compared with the numerous studies done in other parts of the world. Although there are a number of reports regarding diterpenes obtained from propolis around the world, there is no report of propolis comprising diterpenoids from Australia. Propolis is defined as one of the natural rich sources of biologically active diterpenes [12]. Compounds isolated from propolis may be assessed for their medical uses since knowledge of a degree of safety and possible efficacy exists through their traditional consumption as blends in propolis [33,34,35,36,37,38]. With attention to promising biological activities of diterpenes and considering the fact that no reports have been made about diterpenes from Australian propolis, Kangaroo Island propolis samples were documented to possess interesting composition, uncovering novel diterpenes. Chromatography isolation and NMR studies demonstrated that the main components were new serrulatanes, classifying a discrete propolis type, myoporum “purple spot” propolis, with potential for useful medicinal properties. The distinctive “purple spot” was found to originate from compound **1** by its oxidation reaction when exposed to oxygen and light. Based on its ^1^H-NMR spectrum, the “purple spot” has been identified as compound **3**, formed by processes involving oxidation and cyclisation. The purple colour is representative of an ortho-benzoquinone chromophore [39,40]. Multiple propolis samples were analyzed, either collected by research team members or by local beekeepers from different sites over the Island in all seasons. To categorize the samples having the isolated serrulatanes, ^1^H-NMR methods and, to detect the plant source of this propolis type, the bee observation process along with flora distribution mapping were exploited. Bees foraging for propolis were captured from a shrub or tree, of the family Scrophulariaceae, dispersed along the coastal areas, identified as *Myoporum insulare*. 

Matching profiles were confirmed among samples of shiny resinous plant leaves, propolis from bee legs and collected hive propolis using NMR and HPLC analysis. In the case of myoporum propolis samples, diterpenes **1** and **2** were noted as the major diterpenoid components. This is the first report about isolated diterpenes from this Island propolis.

## 4. Materials and Methods

### 4.1. General Experimental Procedures

Thin layer chromatography (TLC) sheets preloaded with silica gel 60-F254 were supplied from Merck (Darmstadt, Germany). Normal-phase short-column vacuum chromatography (NP-SCVC) was done using silica gel 60H (Merck, Darmstadt, Germany) packed into a sintered glass funnel with vacuum applied to give evenly packed stationary phase. A multiband UV-254/366 UV GL-58 mineral light lamp (Analytik Jena, Upland, CA, USA) was used for visualizing the TLC plates. All solvents used were purchased from Chem-Supply Pty Ltd., Port Adelaide, South Australia. The NMR solvents were purchased from Novachem Pty Ltd., Collingwood, Victoria, Australia. To evaporate the solvent fractions, a rotavapor model R-114 rotary evaporator (BÜCHI Labortechnik, Flawil, Switzerland). with a water bath temperature ranging from 40 °C to 60 °C was used. A vacuum pump V700 (Vacuubrand GMBH, Wertheim, Germany)., or Vacuubrand MD 4C NT diaphragm pump (Vacuubrand GMBH, Wertheim, Germany) with vacuum controller V-800 or V-850 were used along with the rotary evaporator and finally a NAPCO 5831 vacuum oven (NAPCO, Salt Lake City, UT, USA) with a Direct Torr vacuum pump (Sargent-Welch, Buffalo, NY, USA) was utilized to remove the last traces of solvents from samples. Analytical HPLC was performed on a Shimadzu UFLC, LC20AD pump, SIL-20A HT auto sampler (Shimadzu, Kyoto, Japan), with a Hewlett Packard Column, NUCLEOSIL 100C18, 5 μm, 4 mm × 125 mm (Shimadzu, Kyoto, Japan). Optical rotation measurements were performed by a Perkin-Elmer Model 341 LC Polarimeter (Perkin-Elmer, Waltham, MA, USA) with a sodium lamp (λ = 589 nm) using a 1 mL cell (length 2.5 cm). UV-vis spectra were measured on a Shimadzu UVmini-1240 spectrophotometer (Shimadzu, Kyoto, Japan). IR spectra were determined with a Shimadzu IRTracer-100 FT-IR spectrometer (Shimadzu, Kyoto, Japan). 1D/2D NMR (Nuclear magnetic resonance) analyses were done with Varian 400 MHz and Bruker 600 MHz systems. NMR spectra were internally referenced to tetramethylsilane (TMS) or 7.27 ppm for the peak of the residual H of CDCl_3_. Low resolution ESI-MS spectra were obtained from a Thermo Finnigan Polarisq ION trap MS/MS system (Thermo Fisher, Waltham, MA, USA). High resolution ESI-MS were measured on a Bruker Apex Qe 7T, Fourier Transform Ion Cyclotron Resonance mass spectrometer (Bruker, Bremen, Germany) equipped with an Apollo MTP ESI/MALDI Duel source (Bruker, Bremen, Germany).

### 4.2. Materials

A large number of standard 10-frame per box hives, each with a propolis mat underneath the hive cover lid, were the source of the propolis samples prepared by a number of beekeepers from colonies of European honey bee (*Apis mellifera ligustica*) in ten apiary sites situated in different locations of Kangaroo Island, South Australia. These sites were in the neighborhood of Hanson Bay (35°58’4” S; 136°49’1” E), Island Beach (35°47’49” S; 137°4745” E), Penneshaw (35°43’6” S; 137°56’25” E), Flour Cask Bay (35°51’35” S; 137°41’44” E), Vivonne Bay (35°59’1” S; 137°10’30” E), Rainy Creek (35°57’8” S; 137°12’55” E), Kingscote (35°39’11” S; 137°38’2” E), Brownlow (35°39’54” S; 137°37’8” E), and Eleanor River (35°57’59” S; 137°12’18” E). These locations are recognized to be a preferred environment for the plant known as *Myoporum insulare* on the Island. These sites delivered 23 propolis samples which were chemically studied, profiled and categorized by TLC analysis and ^1^H-NMR techniques and assessed to be at least 90% sourced from *Myoporum insulare*. Samples were cleaned, packed and deposited at −20 °C until advance analysis. The leaves from *Myoporum insulare* R. Br. were collected from the areas close to the apiary sites located in the Island from December 2013 to June 2016. A specimen was authenticated by the botanist, A/Prof Murray Henwood, John Ray Herbarium, as *Myoporum insulare*. Honey bees observed collecting resin from the leaves of *Myoporum insulare* were captured, capped and frozen in suitable plastic sample tubes. Parts of bee hind legs carrying propolis were cut and stored at −20 °C until analysis.

### 4.3. Extraction and Isolation of Serrulatane Diterpenes from Propolis

Propolis (2 g) was dissolved at room temperature in ethanol (40 mL) using an ultrasound sonicator, then the extract was filtered, and dried using a reduced pressure rotary evaporator followed by a vacuum oven. The dried extract (1.65 g) was taken onto a NP-SCVC column with 5 cm of bed diameter and 4 cm of height. The column was eluted using a stepwise gradient mobile-phase system with 50 mL fractions: hexane (Fractions A_1_ and A_2_); hexane:EtOAc, 4:1 (A_3_ and A_4_); hexane:EtOAc, 1:1 (A_5_ and A_6_); EtOAc (A_7_ and A_8_); EtOAc:EtOH, 4:1 (A_9_ and A_10_). Based on TLC analysis and NMR studies results, fractions A_4_ and A_5_ were combined and designated as fraction B. Exploiting the same technique, fractions A_6_ and A_7_ were combined together to give fraction B’. Fraction B (692 mg) was eluted using the stepwise gradient mobile-phase system hexane reaching hexane:EtOAc 4:1 and finishing with hexane: EtOAc 1:1. Totally 10 fractions (B_1–10_) were collected and fractions B_3–5_ were grouped together and chosen based on TLC and NMR analysis for further purification using NP-SCVC with CH_2_Cl_2_ and stepwise increasing the proportion of EtOAc to 1%, 2%, 4% and finally to 8% as eluent, ten fractions, C_1–10_, were collected of which fraction C_5_ provided compound **2** (153 mg). Further fractionation of fraction B’ (758 mg) was completed by NP-SCVC with CH_2_Cl_2_ and stepwise increasing the proportion of EtOAc to 2%, 4%, 8% and finally to 20% as mobile phase that afforded to 10 fractions (B’_1–10_). Through TLC and NMR analysis fraction B’_7_ was kept for one more purification step and fractions B’_6_ and B’_8_ were mixed together and called fraction D’. Fraction B’_7_ (86 mg) was loaded on NP-SCVC with CH_2_Cl_2_ and stepwise increasing the proportion of EtOAc to 1%, 2%, 4%, 8%, 12% and finally to 16% as eluent which resulted in 13 fractions (C’_1–13_) and fractions (C’_11_ and C’_12_) gave compound **1** (41 mg). Fraction D’ (249 mg) was further fractionated using NP-SCVC with CH_2_Cl_2_ and stepwise increasing the proportion of EtOAc to 1%, 2%, 4%, 8%, 12% and finally to 16% as mobile phase which gave 13 fractions (D’_1–13_) and fractions D’_8–11_ yielded compound **3** (62 mg).

Compound **1**, C_20_H_30_O_3_, 7,8,18-trihydroxyserrulat-14-ene. Yellow oily material. [α]${}_{\text{D}}^{20}$ −27.3° (*C* = 1.0, CHCl_3_). UV (CH_3_OH) λ_max_ nm (log ε) 279 (3.37) and 320 (2.49). IR (ν): 3381, 2924, 2864, 1625, 1581, 1448 cm^−1^. For ^1^H- & ^13^C-NMR, see Table 1 and Table 2. HRESIMS: *m*/*z* 341.2089 [M + Na]^+^ (calcd. 341.2087).

Compound **2**, C_20_H_28_O_3_, (18*RS*)-5,18-epoxyserrulat-14-en-8,18-diol. Dark red oily material. [α]${}_{\text{D}}^{20}$ −21.5° (*C* = 1.0, CHCl_3_). UV (CH_3_OH) λ_max_ nm (log ε) 293 (3.36) and 334 (2.91). IR (ν): 3327, 2970, 2926, 2860, 1647, 1558, 1448, 1043 cm^−1^. For ^1^H- & ^13^C-NMR, see Table 1 and Table 2. HRESIMS: *m*/*z* 315.1970 [M − H]^−^ (calcd. 315.1966).

Compound **3**, C_20_H_26_O_3_, 5,18-epoxyserrulat-14-en-7,8-dione. Purple oily material. For ^1^H- & ^13^C-NMR, see Table 1 and Table 2. HRESIMS: *m*/*z* 315.1957 [M + H]^+^ (calcd. 315.1955).

### 4.4. Acetylation of Serrulatane Diterpenes

The serrulatane **1** (400 mg) was dissolved in acetic anhydride (1 mL) and dry pyridine (1 mL) and stirred at room temperature overnight [41]. The mixture was quenched with distilled water (30 mL), and was extracted with CH_2_Cl_2_. The CH_2_Cl_2_ solution was extracted with 0.1 M hydrochloric acid (30 mL) to remove the residual trace of pyridine, and the aqueous phase was extracted using CH_2_Cl_2_ (3 × 40 mL). The CH_2_Cl_2_ solutions were dried over Na_2_SO_4_, filtered and concerted on a rotary evaporator to give a yellow oily residue. Finally, using short-column vacuum chromatography with CH_2_Cl_2_ and EtOAc (100:0 to 90:10) the compound **4** (434 mg, 77%) was purified and isolated as a yellow viscous liquid. The serrulatane **2** (80 mg) was dissolved in acetic anhydride (1 mL) and in dry pyridine (1 mL) and stirred at room temperature overnight, and with the same workup procedure as for preparation of **4** and using short-column vacuum chromatography with hexane and EtOAc (100:0 to 80:20) compound **5** (57.8 mg, 58%) was purified and isolated. 

Compound **4**, C_26_H_36_O_6_. 7,8,18-triacetoxyserrulat-14-ene. Pale yellow oily material. For ^1^H- & ^13^C-NMR, see Table 1 and Table 2. HRESIMS: *m*/*z* 467.2405 [M + Na]^+^ (calcd. 467.2404).

Compound **5**, C_24_H_32_O_5_, (18*S*)-5,18-epoxyserrulat-14-en-8,18-dioate. Pale yellow oily material. For ^1^H- & ^13^C-NMR, see Table 1 and Table 2. HRESIMS: *m*/*z* 423.2143 [M + Na]^+^ (calcd. 423.2142).

## Figures and Tables

**Table 1:** ^1^H-NMR data for Compounds **1**–**5** (400 MHz, δ in ppm *J* values (Hz) in parentheses, measured in CDCl_3_ relative to tetramethylsilane (TMS)).

#H	1	2	3	4	5
1	3.07 *pd* (6.8, 1.6)	3.11 *sextet* (7.6)	2.82 *m*	2.92 *m*	2.96 *sextet* (7.4)
2	A: 1.51 *m* B: 1.99 *m*	A: 2.25 *m* B: 1.42 *m*	A: 2.15 *m* B: 1.23 *m*	A: 1.87 *m* B: 1.50 *m*	A: 2.19 *m* B: 1.42 *m*
3	A: 1.66 *m* B: 1.86 *m*	A: 2.08 *m* B: 1.05 *m*	A: 2.13 *m* B: 1.11 *m*	A: 1.87 *m* B:1.60 *m*	A: 2.12 *m* B: 1.10 *m*
4	2.76 *td* (5.6, 2.6)	2.54 *td* (11.4, 3.6)	2.14 *m*	2.88 *m*	2.54 *td* (11.6, 3.8)
5	6.56 *s*	-	-	6.92 *s*	-
6	-	-	-	-	-
7	-	6.48 *s*	-	-	6.70 *s*
8	-	-	-	-	-
9	-	-	-	-	-
10	-	-	-	-	-
11	1.83 *m*	1.52 *m*	1.74 *m*	2.12 *m*	1.65 *m*
12	A: 1.36 *m* B: 1.25 *m*	A: 1.69 *m* B: 1.27 *m*	A: 1.19 *m* B: 1.76 *m*	A: 1.33 *m* B: 1.23 *m*	A: 1.73 *m* B: 1.30 *m*
13	A: 1.86 *m* B: 1.90 *m*	A: 2.20 *m* B: 2.09 *m*	A: 2.09 *m* B: 1.97 *m*	A: 1.99 *m* B: 1.87 *m*	A: 2.19 *m* B: 2.02 *m*
14	5.04 *bt* (7.0)	5.14 *bt* (7.0)	5.07 *bt* (6.8)	4.97 *bt* (7.1)	5.08 *bt* (6.5)
15	-	-	-	-	-
16	1.68 *s*	1.70 *s*	1.71 *s*	1.66 *s*	1.69 *s*
17	1. 58 *s*	1. 63 *s*	1.63 *s*	1. 54 *s*	1.62 *s*
18	3.63 *d* (6.2)	5.59 *bs*	A: 3.86 *t* (10.8) B: 4.49 *dd* (10.8, 3.6)	4.11 *d* (6.3)	6.53 *d* (1.7)
19	2.2 *s*	2.13 *s*	1.81 *s*	2.14 *s*	2.14 *s*
20	1.21 *d* (7.0)	1.28 *d* (6.9)	1.13 *d* (6.9)	1.15 *d* (7.0)	1.23 *d* (6.8)
21	-	-	-	-	-
22	-	-	-	2.06 *s*	2.09 *s*
23	-	-	-	-	-
24	-	-	-	2.29 *s*	2.30 *s*
25	-	-	-	-	-
26	-	-	-	2.31 *s*	-

**Table 2:** ^13^C-NMR data for Compounds **1**–**5** (100 MHz, δ in ppm, measured in CDCl_3_ relative to TMS).

#C	1	2	3	4	5
1	26.9	27.8	28.7	27.6	28.3
2	26.5	31.7	30.8	26.9	31.4
3	20.5	26.6	25.3	19.2	26.2
4	37.9	31.9	40.1	36.9	32.3
5	122.0	141.7	162.8	128.1	145.6
6	121.3	122.9	114.4	128.3	123.9
7	139.7	115.9	179.4	139.3	122.5
8	141.6	146.8	181.9	140.6	142.4
9	127.2	125.2	139.4	134.1	130.9
10	130.7	124.3	149.7	137.1	123.8
11	45.4	40.5	37.1	41.9	38.9
12	29.8	28.9	29.1	28.3	28.3
13	26.2	25.4	24.8	26.1	25.1
14	124.4	124.1	123.1	123.9	123.5
15	131.7	131.9	132.9	132.0	132.6
16	25.7	25.7	25.7	25.7	25.7
17	17.7	17.7	17.8	17.6	17.7
18	64.4	91.9	71.8	65.4	90.2
19	15.5	15.9	7.6	16.1	15.9
20	21.1	22.9	21.7	21.4	22.8
21	-	-	-	171.2	170.2
22	-	-	-	20.9	21.1
23	-	-	-	168.1	170.0
24	-	-	-	20.4	21.2
25	-	-	-	168.3	-
26	-	-	-	20.5	-

## Supplementary Materials

Supplementary materials are available online. S1: NMR and HRESIMS spectra of diterpenes, Video S2: bees collecting resin from the leaves of *Myoporum insulare*.
