# Supplementary material for: Structure Elucidation and Botanical Characterization of Diterpenes from a Specific Type of Bee Glue
Source: Molecules. 2017 Jul 14;22(7):1185. doi: 10.3390/molecules22071185 (PMC6152305; doi:10.3390/molecules22071185)

Structure Elucidation and Botanical Characterization of Diterpenes from a Specific Type of Bee Glue

Noushin Aminimoghadamfarouj and Alireza Nematollahi *

Faculty of Pharmacy A15, The University of Sydney, Sydney NSW 2006, Australia; nami1357@uni.sydney.edu.au (N.A)
*Correspondence: anem7250@uni.sydney.edu.au; Tel.: +61-2-9351-6528; Fax: +61-2-9351-4391

Supporting Information

Table of contents

Compound 1- ^{1}H NMR - 2 -
Compound 1- ^{13}C NMR - 3 -
Compound 1- ^{1}H-^{1}H COSY - 4 -
Compound 2- ^{1}H NMR - 5 -
Compound 2- ^{13}C NMR - 6 -
Compound 2- ^{1}H-^{1}H COSY - 7 -
Compound 3- ^{1}H NMR - 8 -
Compound 3- ^{13}C NMR - 9 -
Compound 3- ^{1}H-^{1}H COSY - 10 -
Compound 4- ^{1}H NMR - 11 -
Compound 4- ^{13}C NMR - 12 -
Compound 4- ^{1}H-^{1}H COSY - 13 -
Compound 4- ^{1}H-^{1}H ROESY - 14 -
Compound 5- ^{1}H NMR - 15 -
Compound 5- ^{13}C NMR - 16 -
Compound 5- ^{1}H-^{1}H COSY - 17 -
Compound 1 - 18 -
Compound 2 - 19 -
Compound 3 - 20 -
Compound 4 - 21 -
Compound 5 - 22 -
Schematic formation of compound 3 - 23 -

Compound **1**- ^{1}H NMR

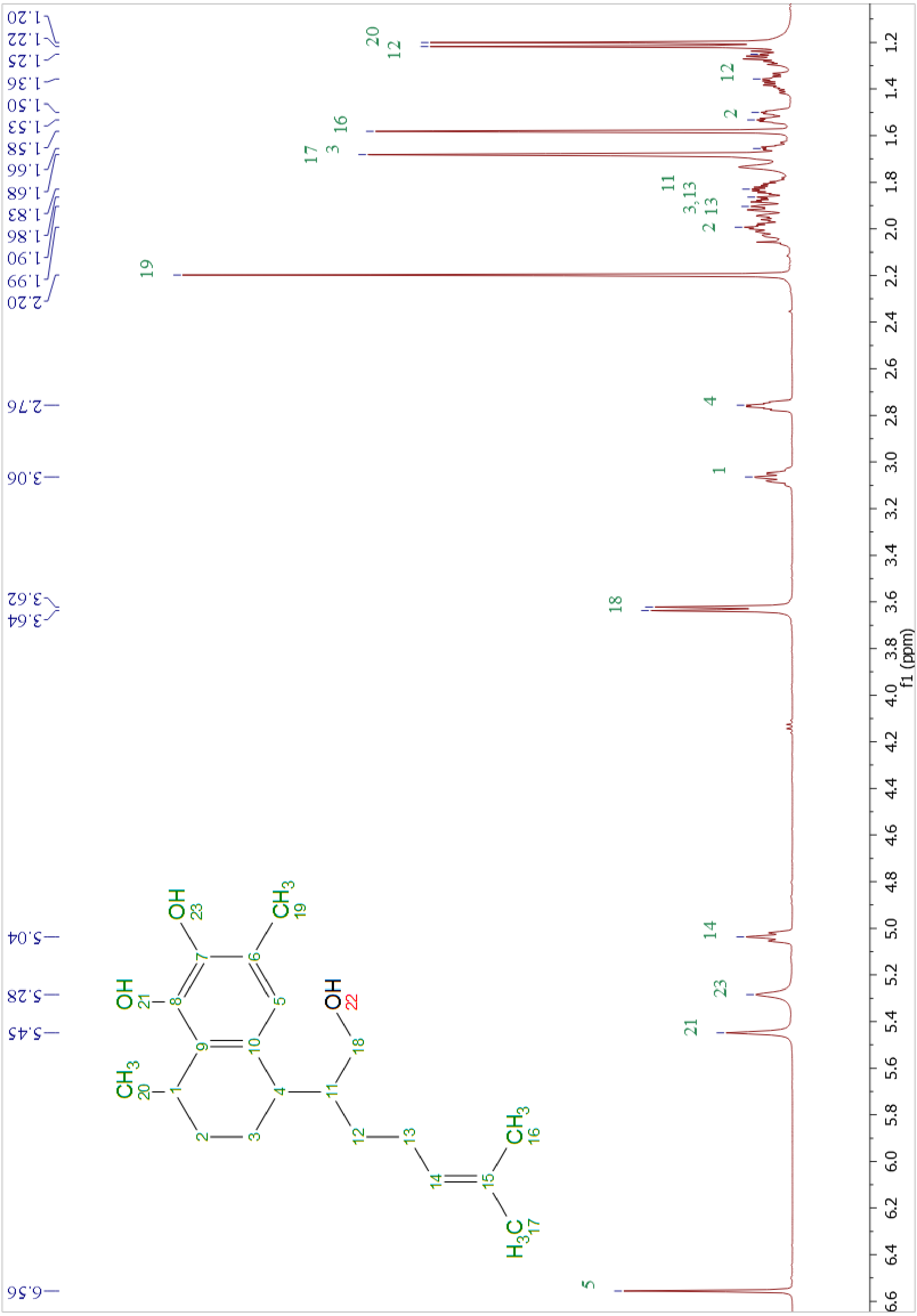

Compound **1**- ^{13}C NMR

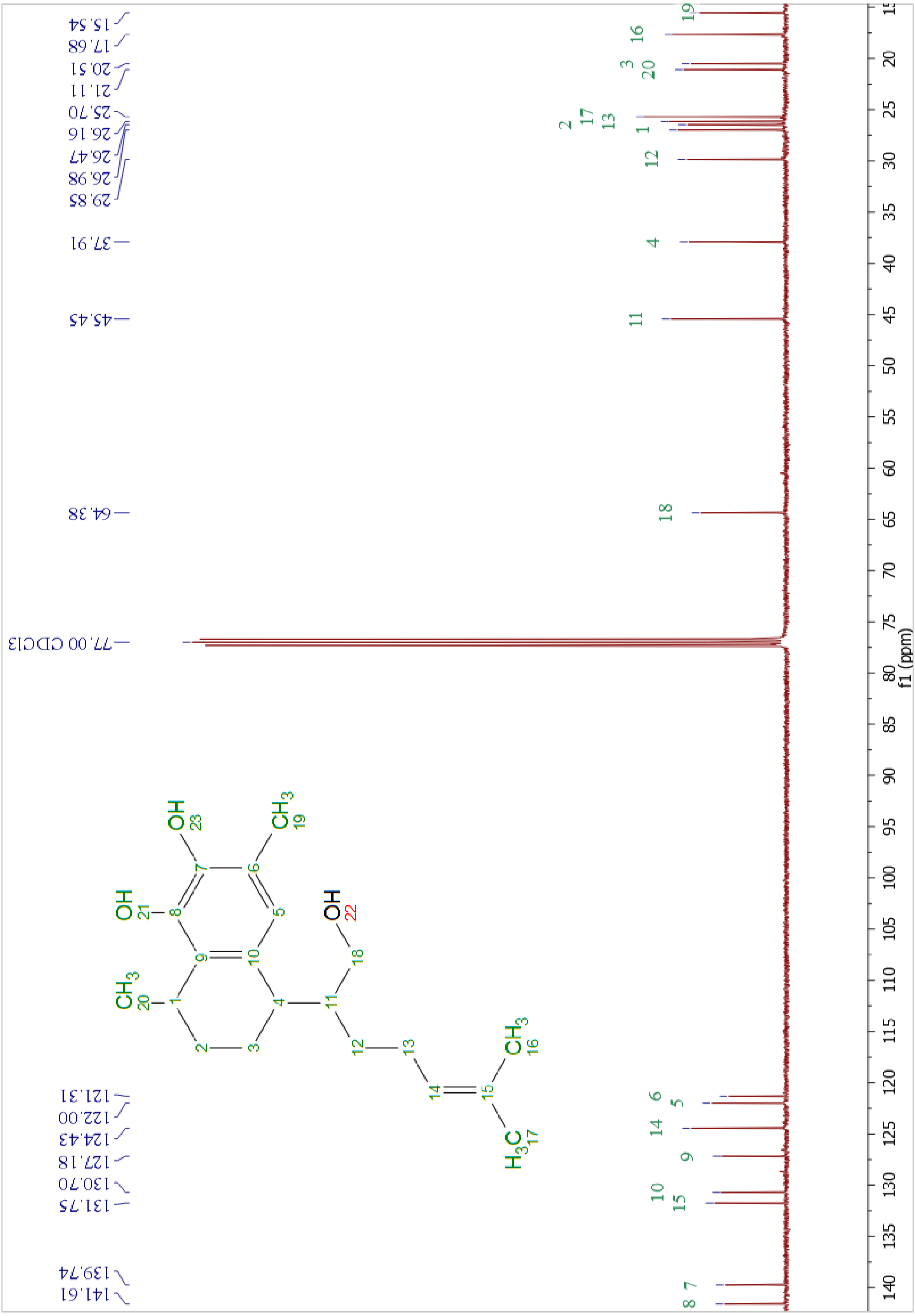

Compound **1**- ^{1}H-^{1}H COSY

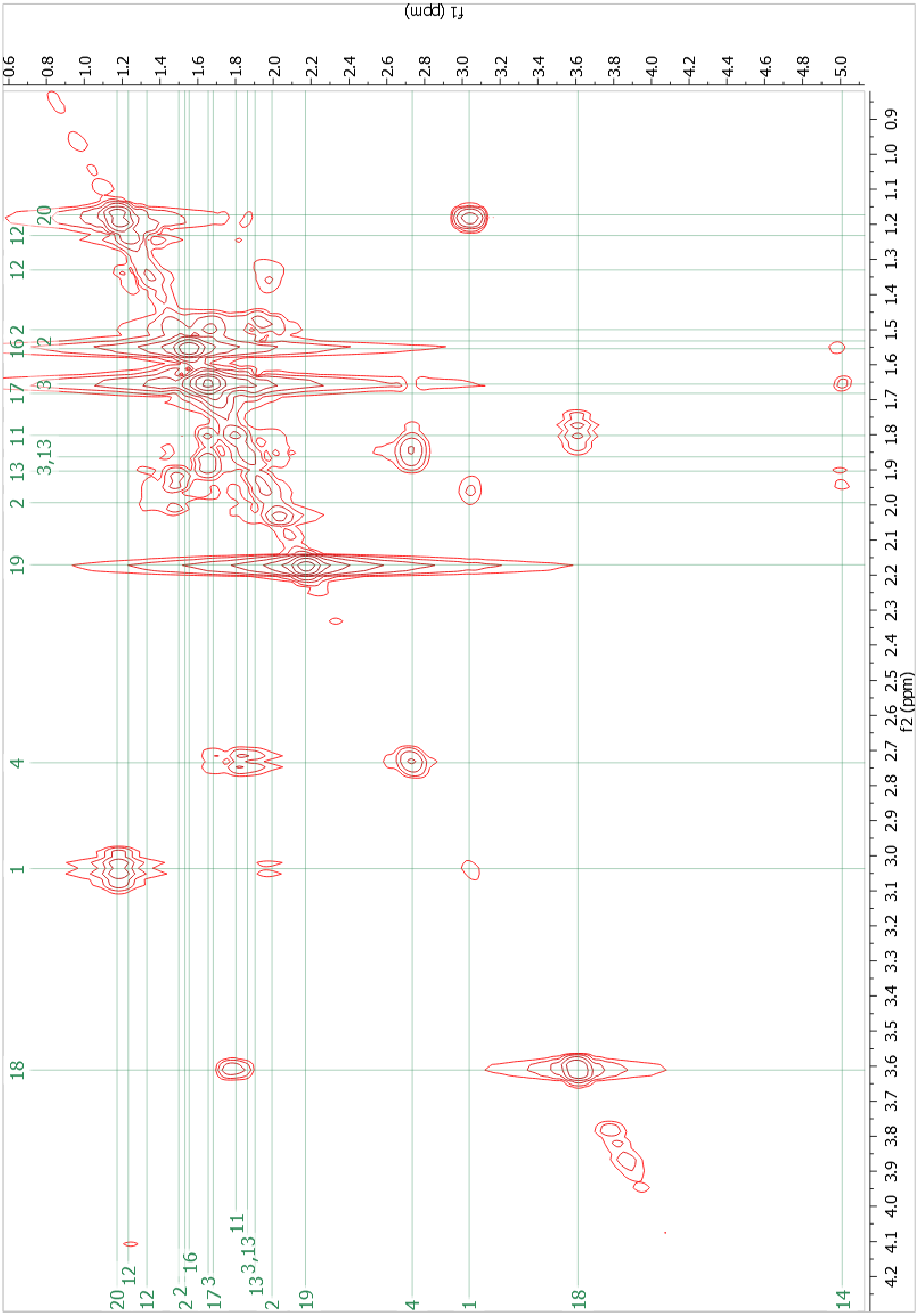

Compound **2**- ^{1}H NMR

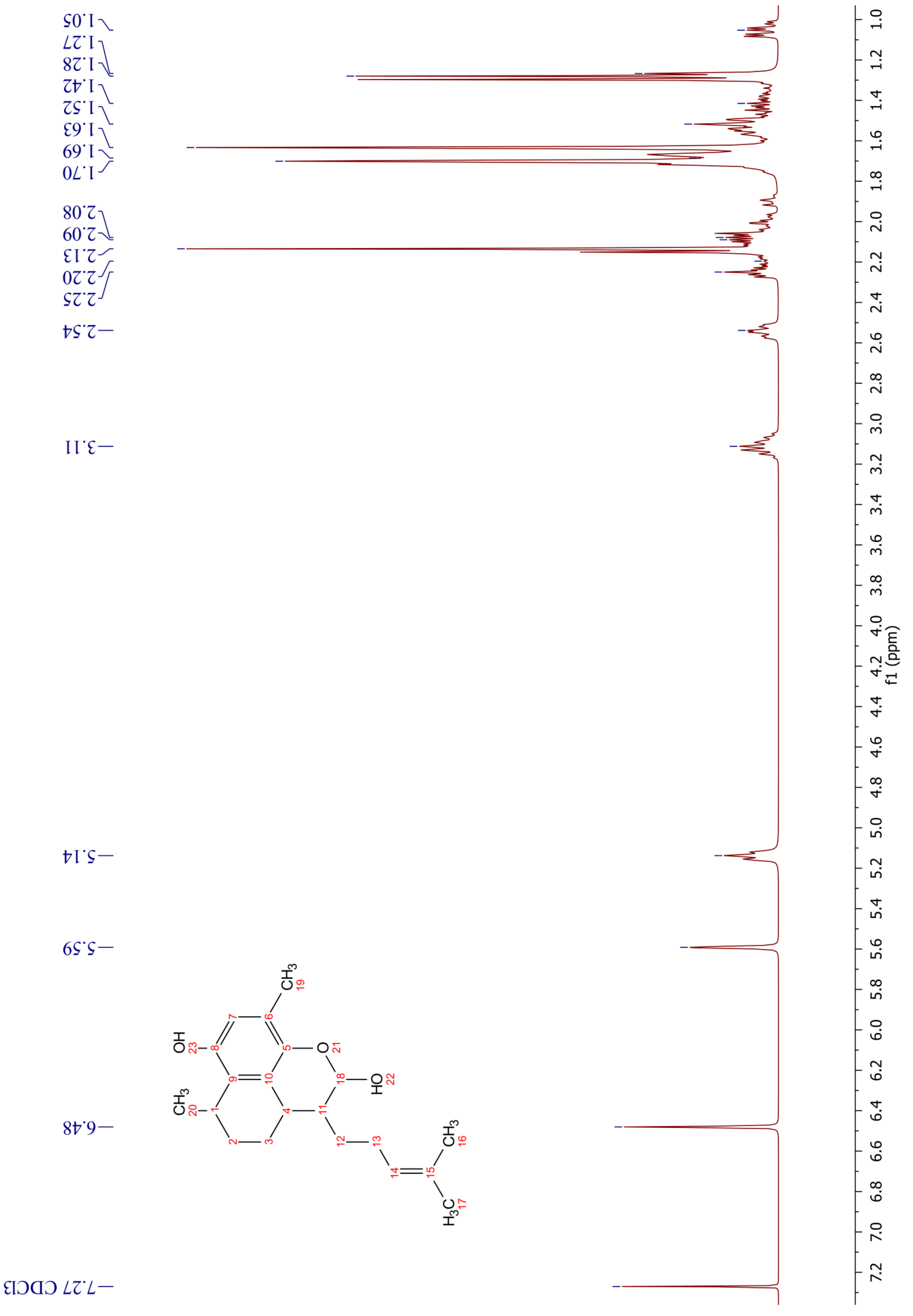

Compound **2**- ^{13}C NMR

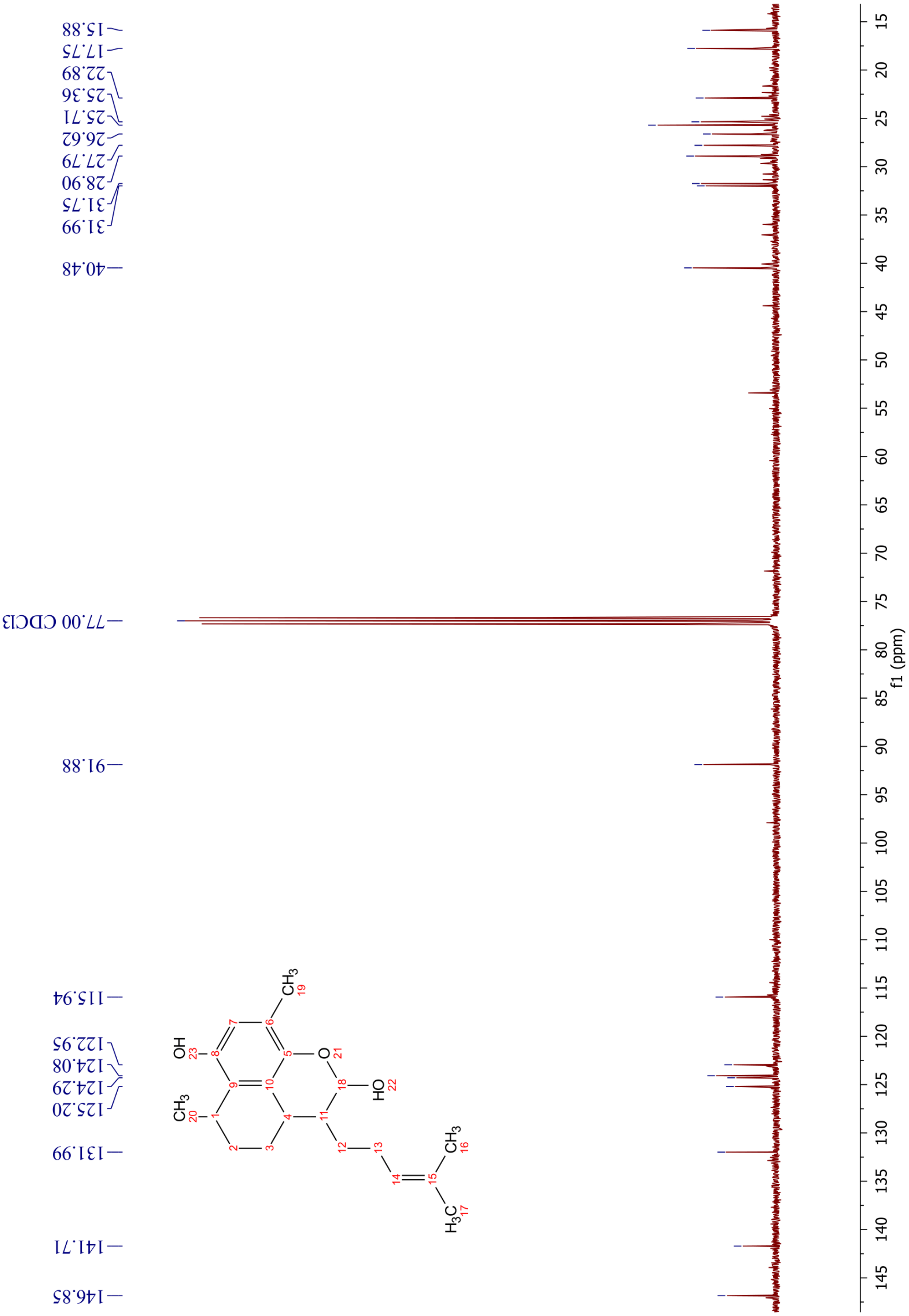

Compound **2**- ^{1}H-^{1}H COSY

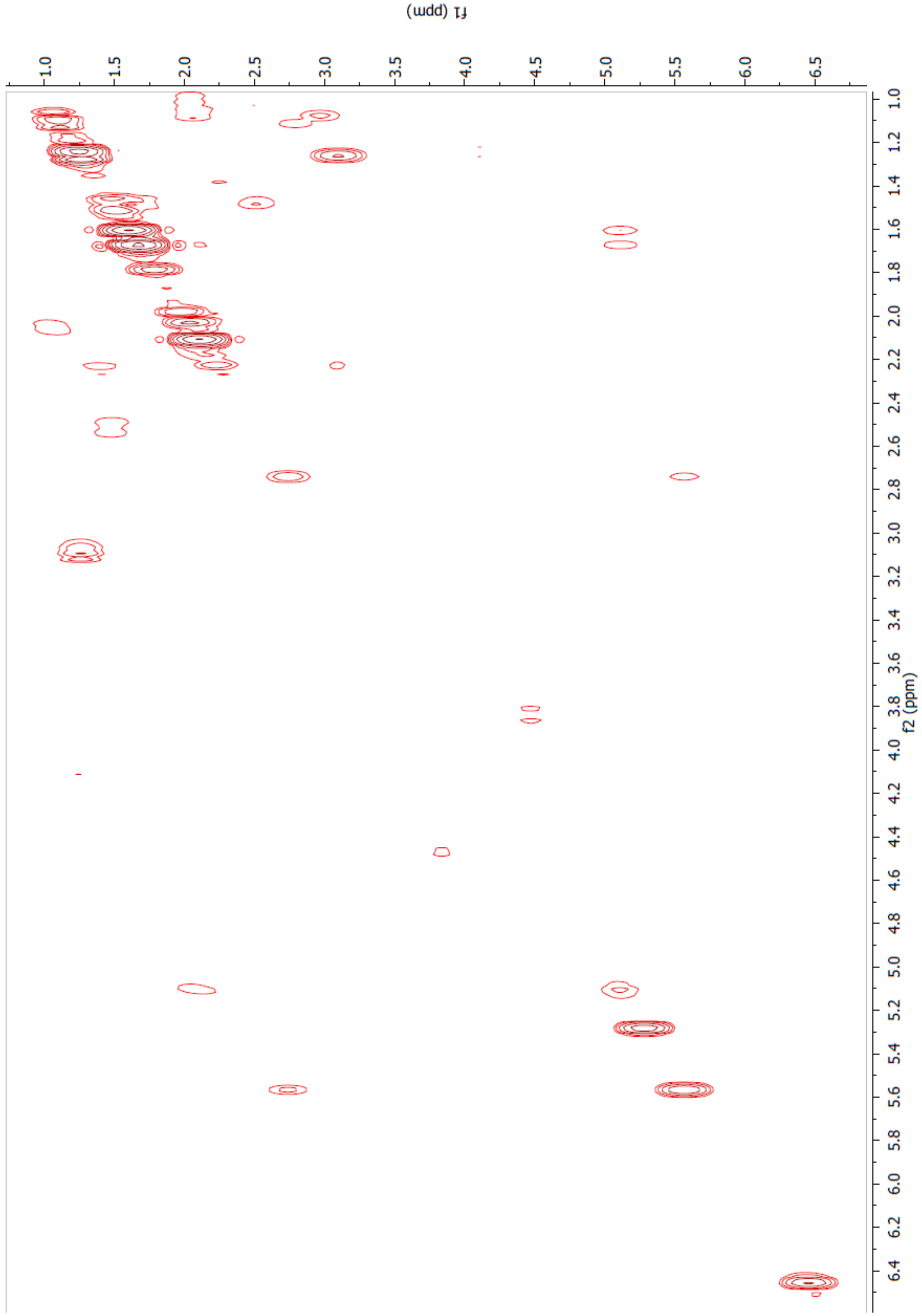

Compound **3**- ^{1}H NMR

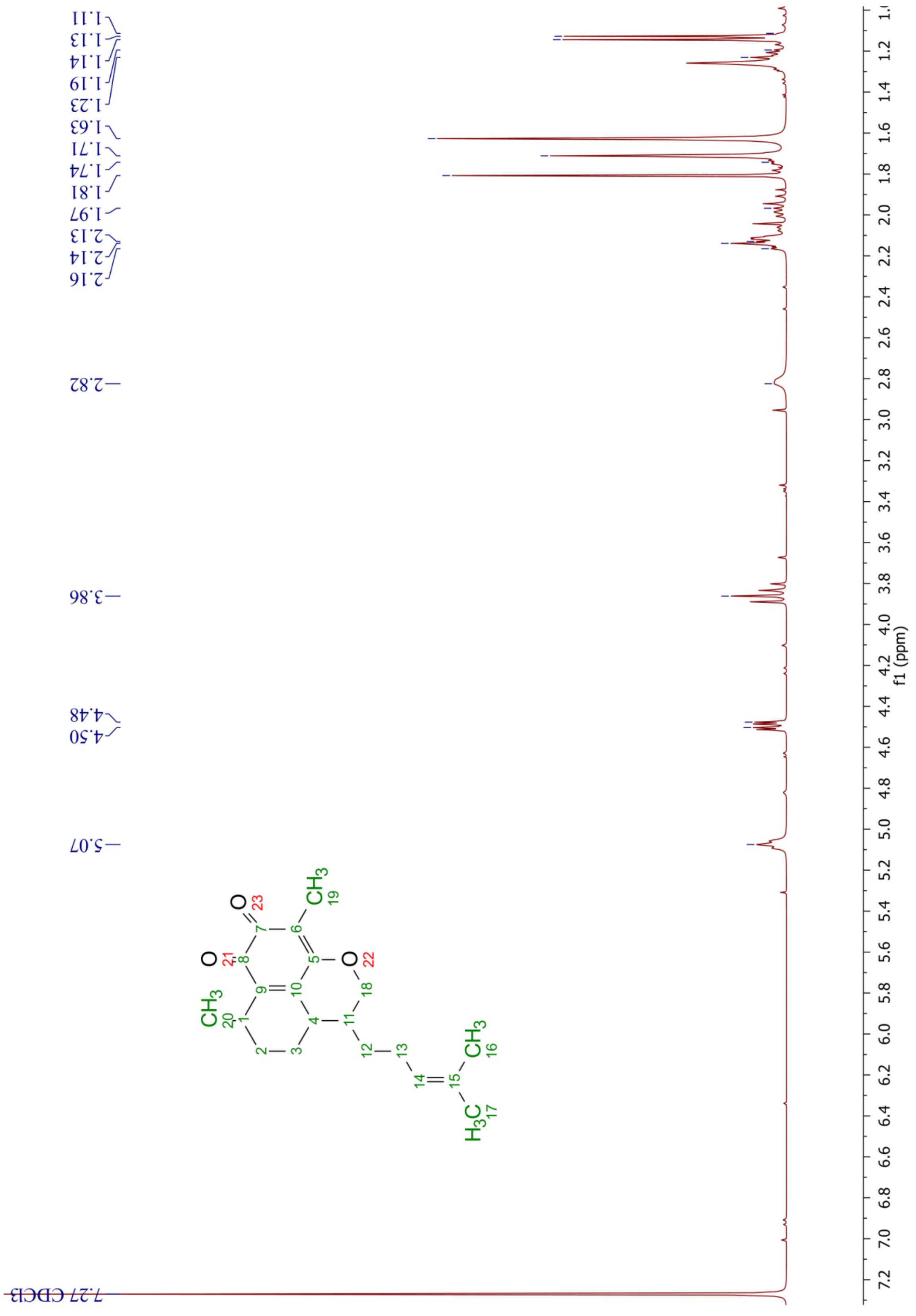

Compound **3**- ^{13}C NMR

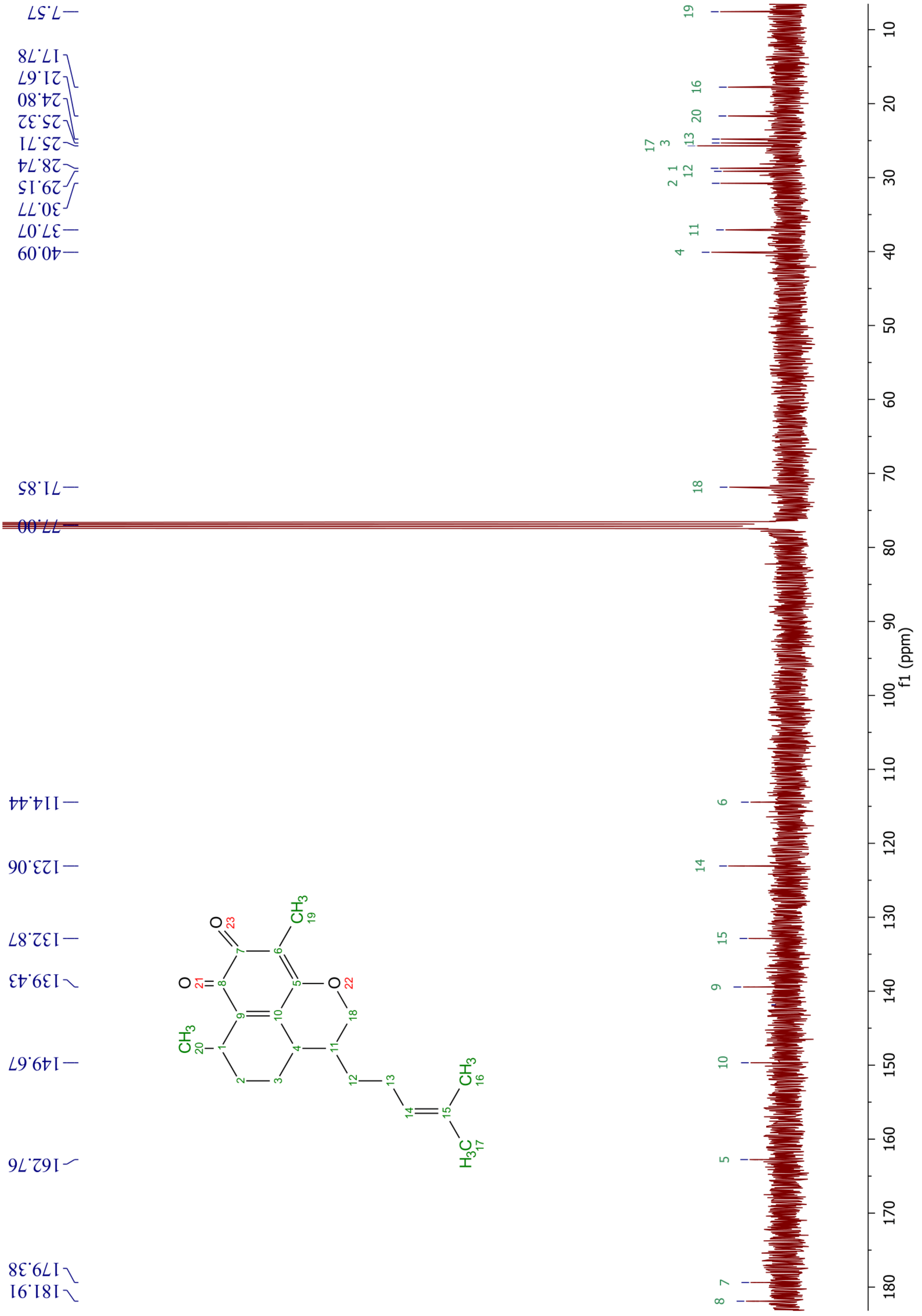

Compound **3**- ^{1}H-^{1}H COSY

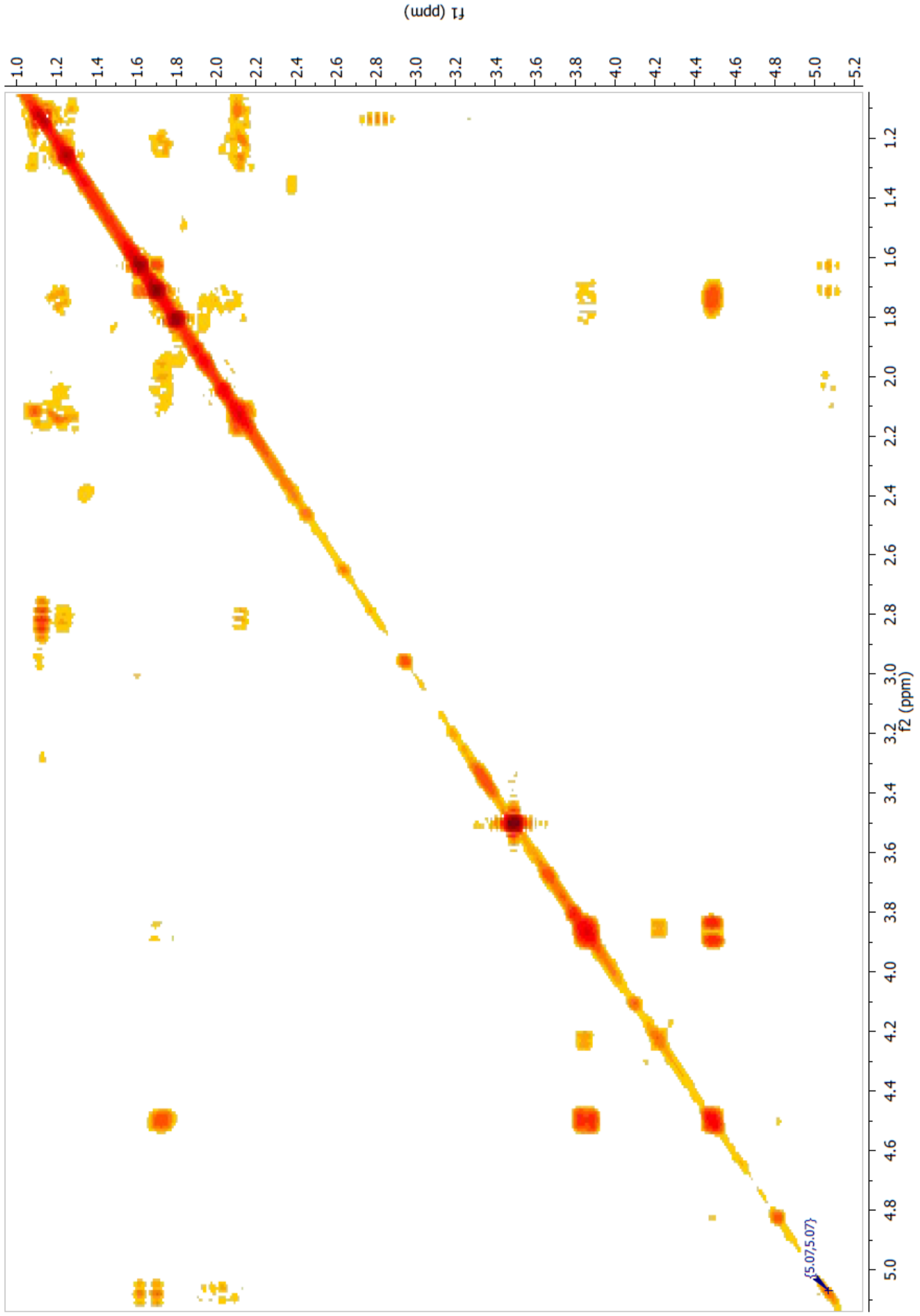

Compound **4**- ^{1}H NMR

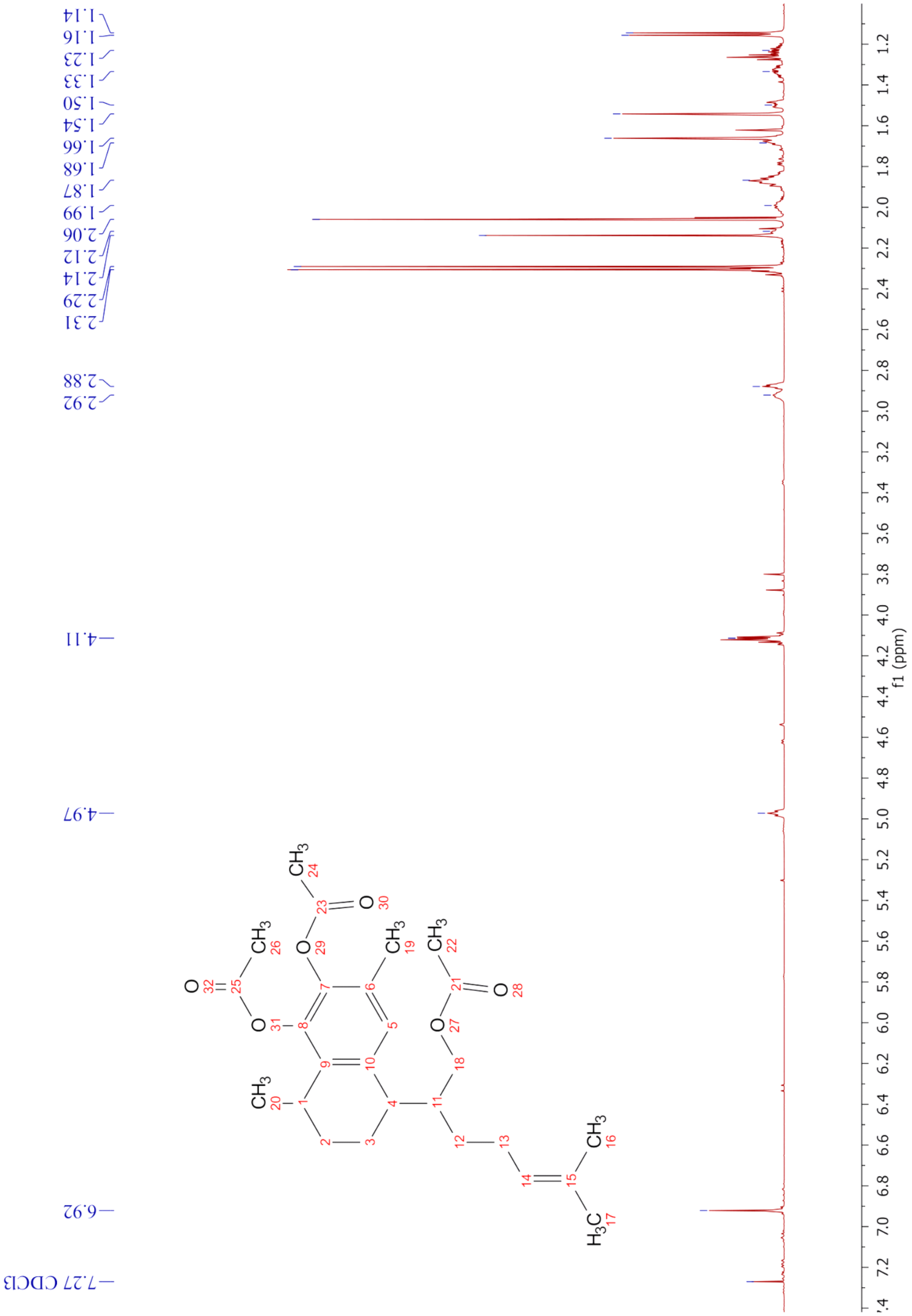

Compound **4**- ^{13}C NMR

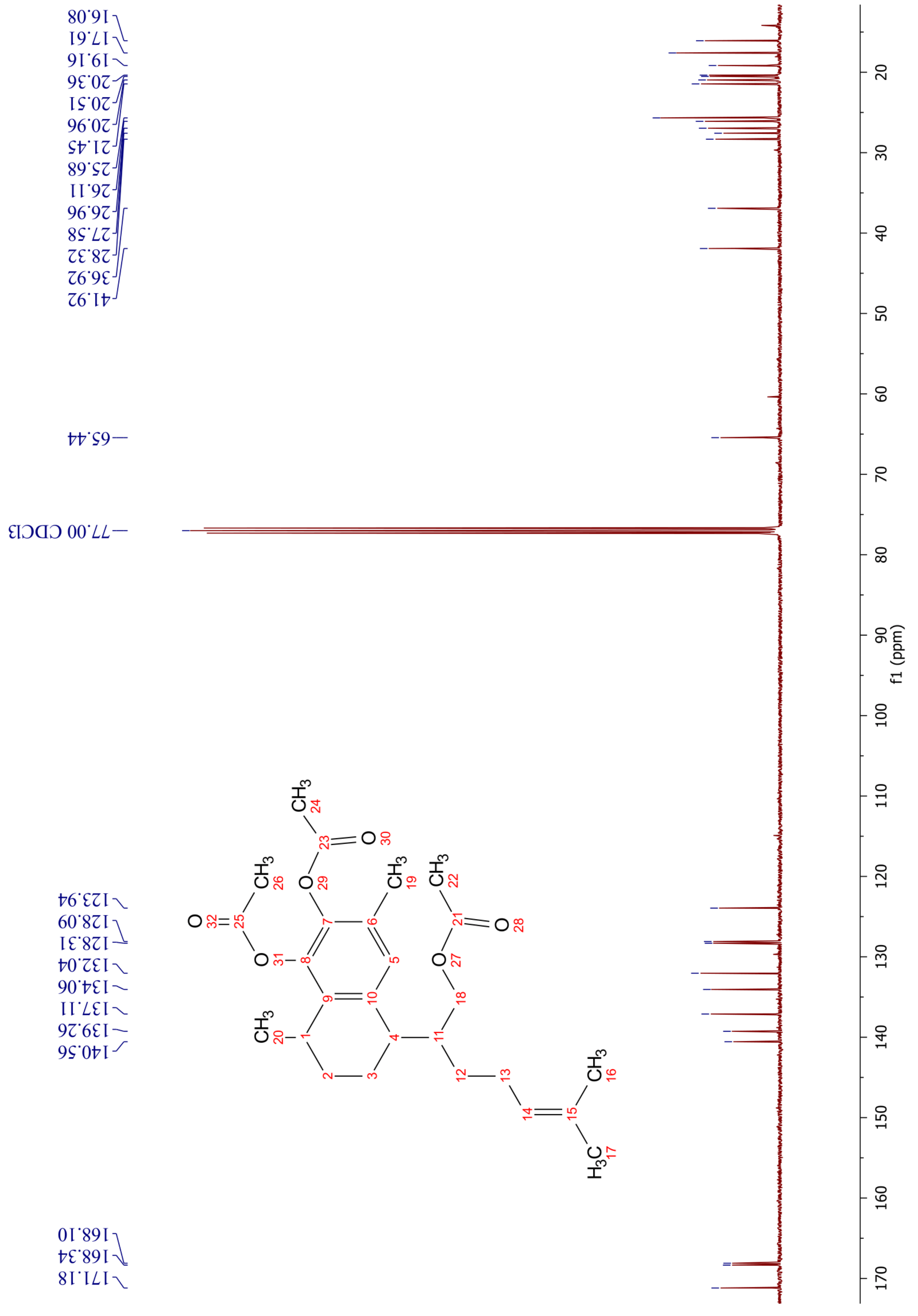

Compound **4**- ^{1}H-^{1}H COSY

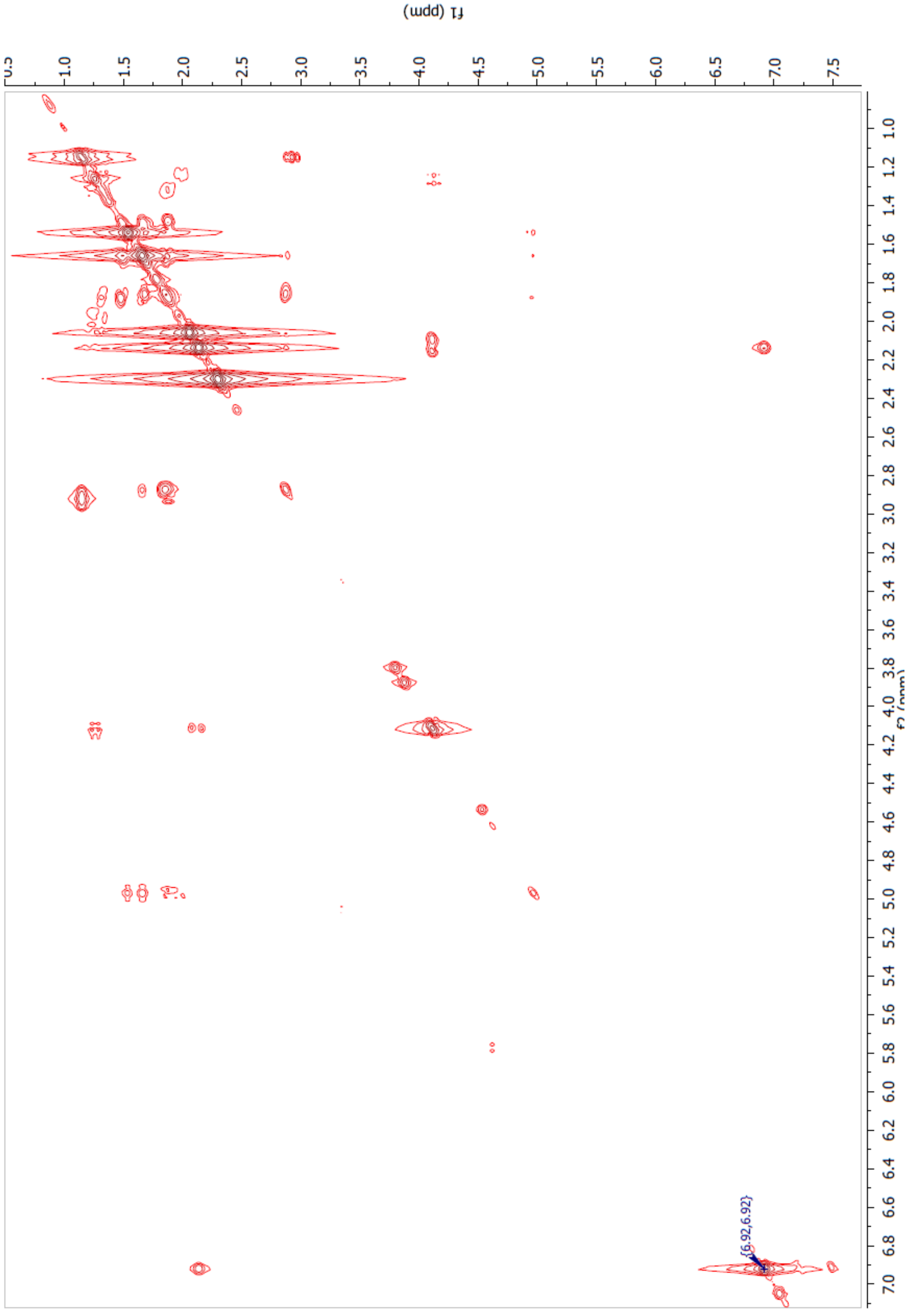

Compound **4**- ^{1}H-^{1}H ROESY

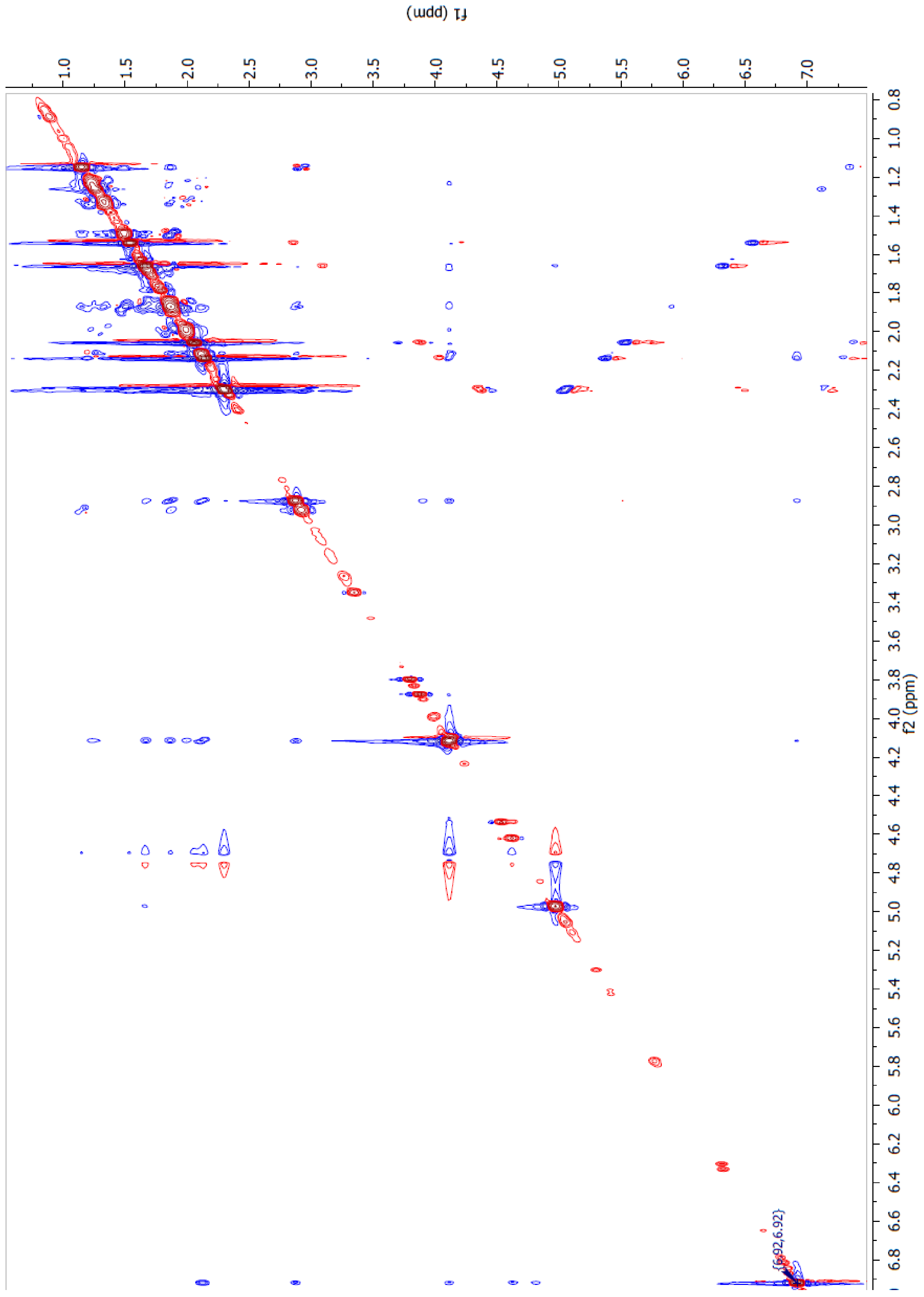

Compound **5**- ^{1}H NMR

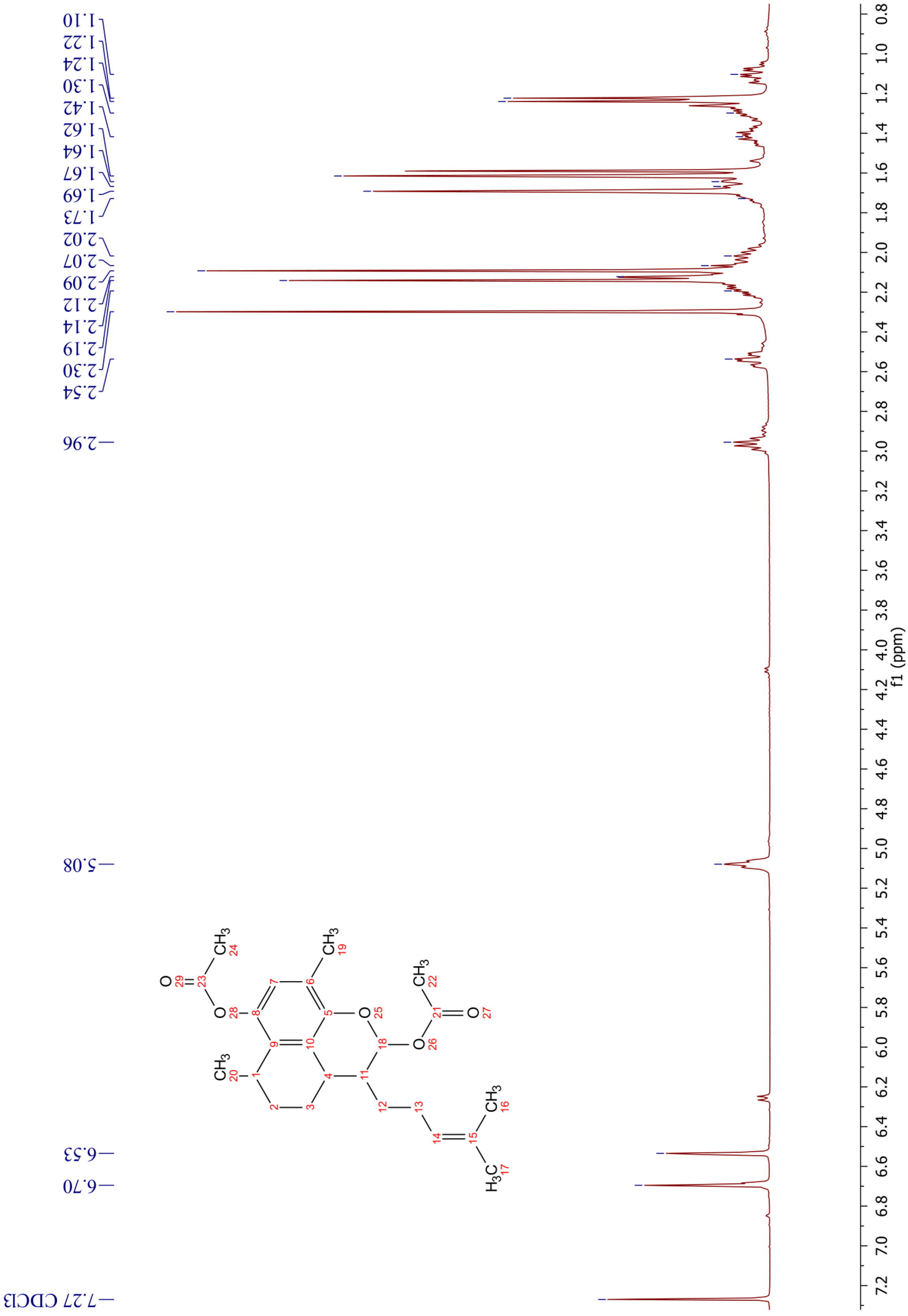

Compound **5**- ^{13}C NMR

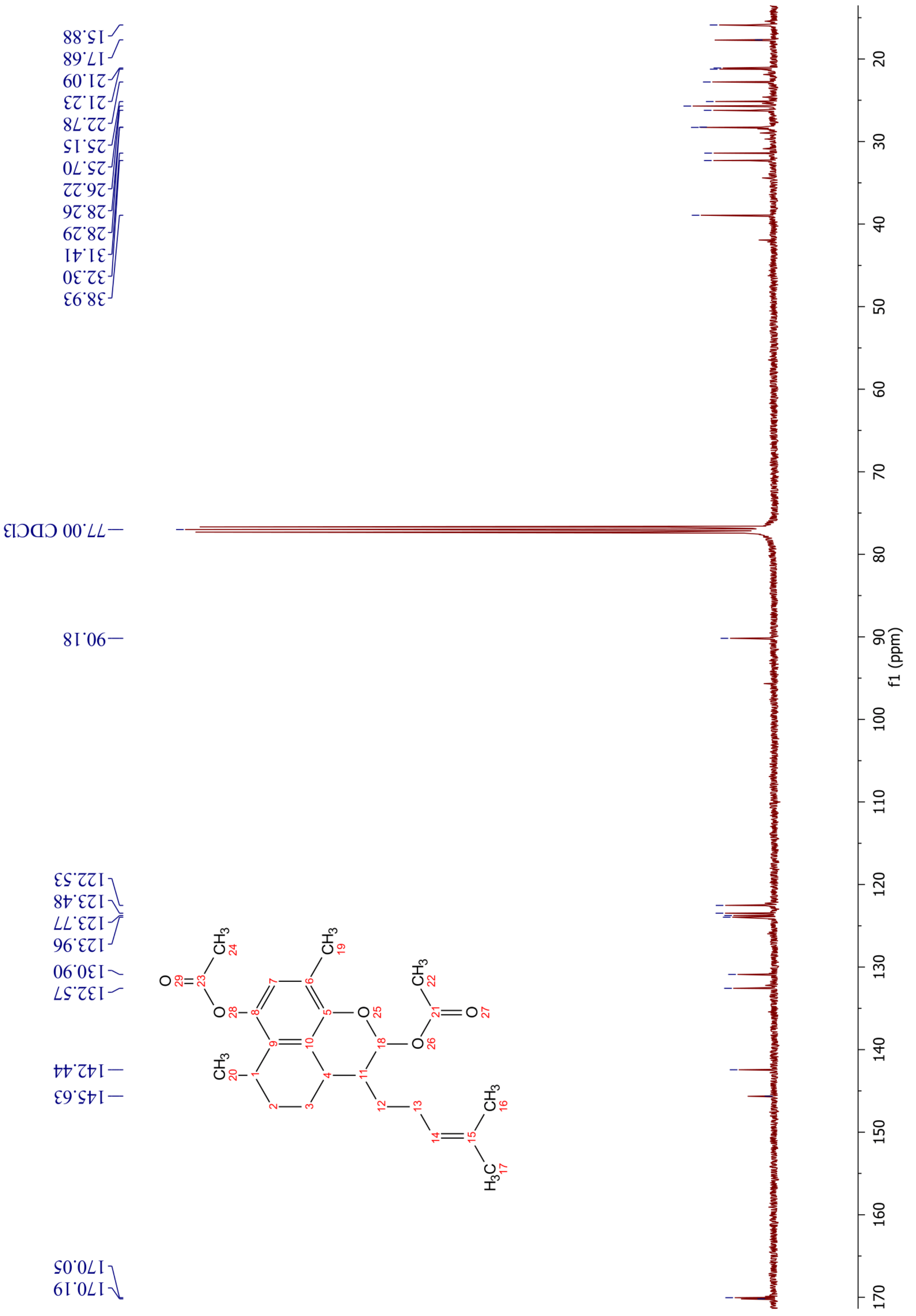

Compound **5**- ^{1}H-^{1}H COSY

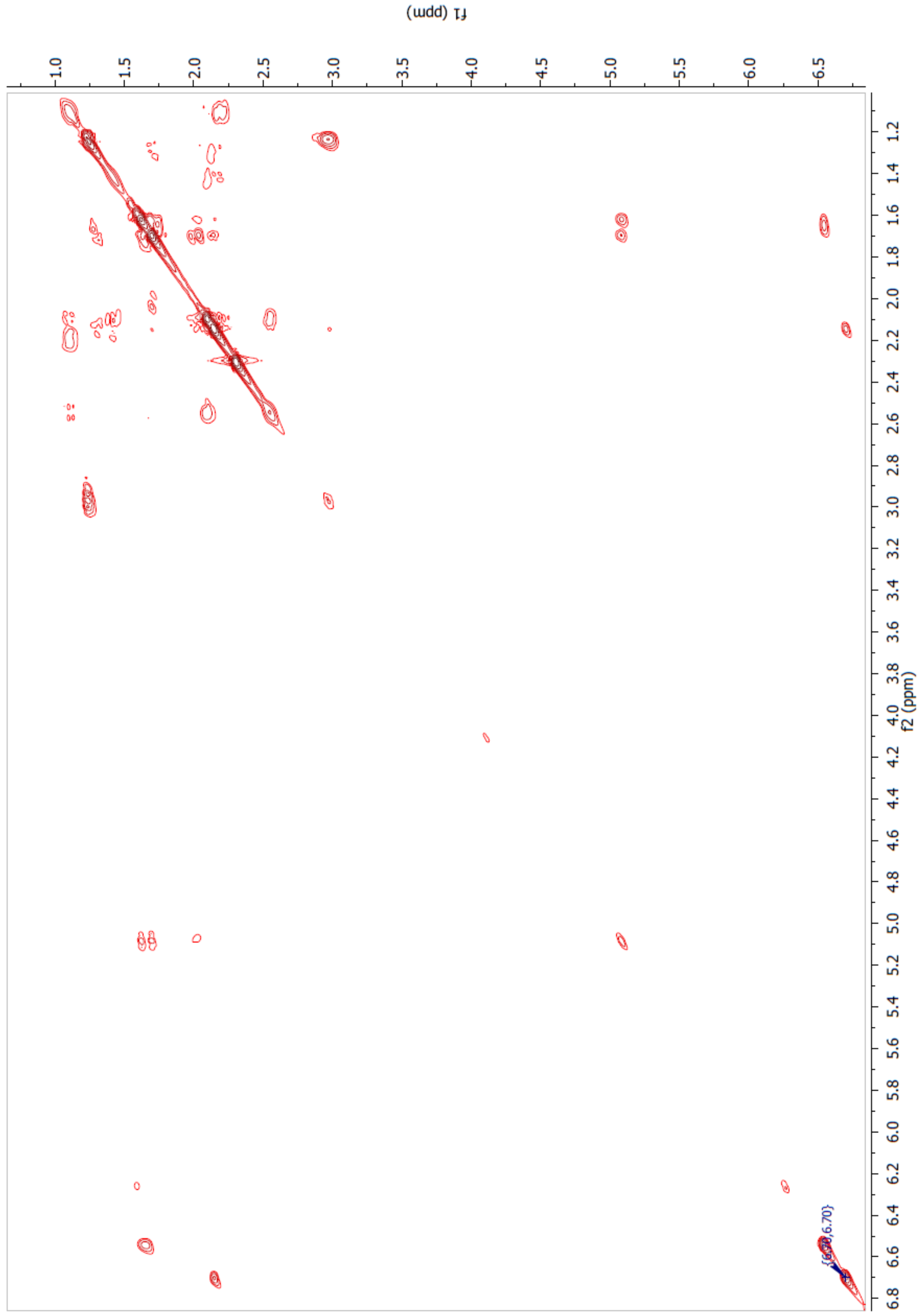

Compound 1

Generic Display Report

Analysis Info

		Acquisition Date	16/10/2014 10:48:13 AM
Analysis Name	D:\Data\Nick-2014-files\ESI_Positive\16-October\2014-10-16\2014-10-16-posesi-service_000006.d		
Method	1MW Positive ESI	Operator	
Sample Name	Compound A	Instrument	apex-Ultra
Comment	MeOH 1 1M TOF delay 0.0006s, Q1 200 m/z		

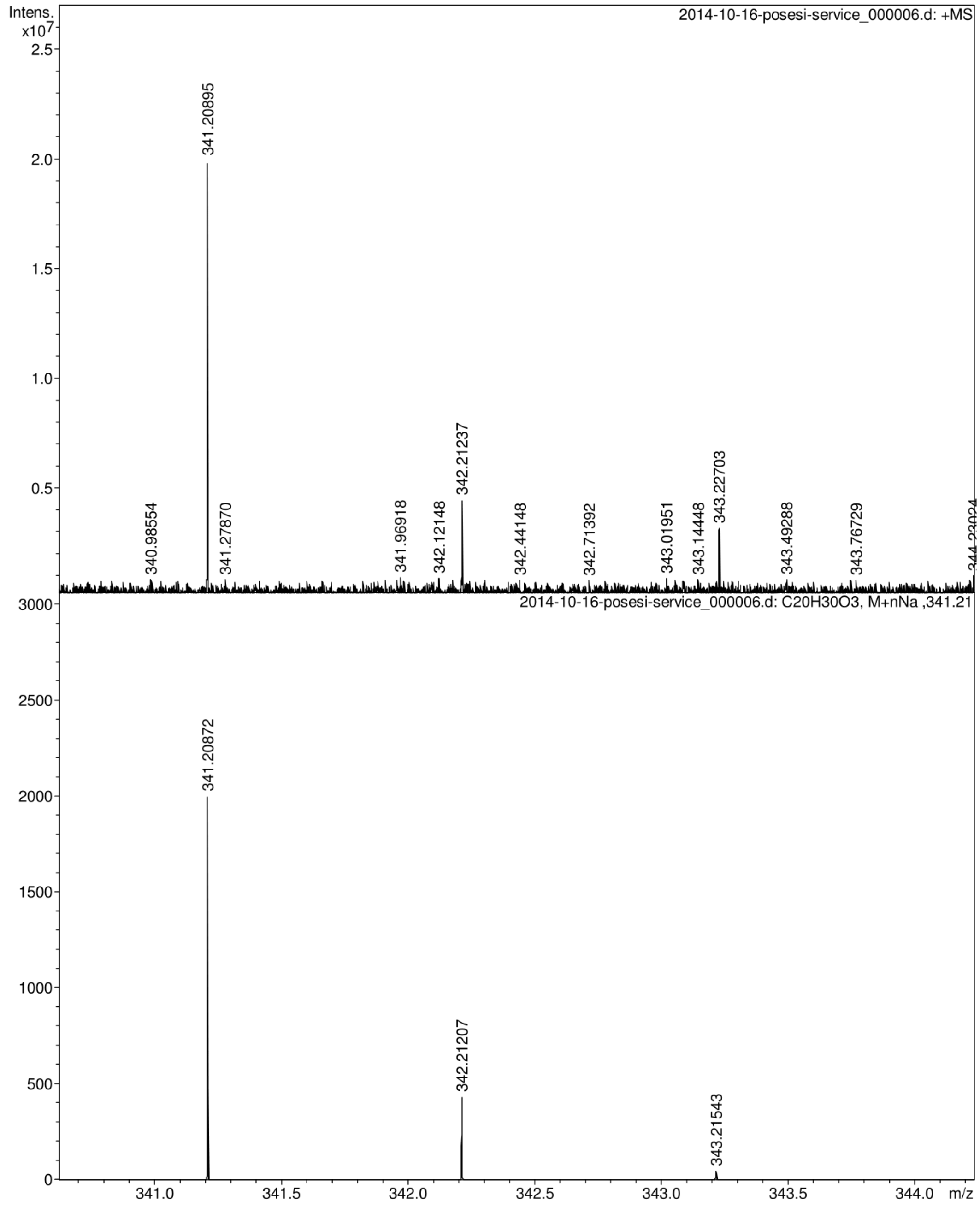

Bruker Compass DataAnalysis 4.0 printed: 16/10/2014 10:52:16 AM Page 1 of 1

Compound 2

Generic Display Report

Analysis Info

Analysis Name	D:\Data\Nick-2014-files\ESI_Negative\10-October\2014-10-22\2014-10-22-esi-neg_000008.d	Acquisition Date	22/10/2014 2:49:42 PM
Method	1MW Positive ESI	Operator	
Sample Name	Compound B	Instrument	apex-Ultra
Comment	MeOH/DCM		

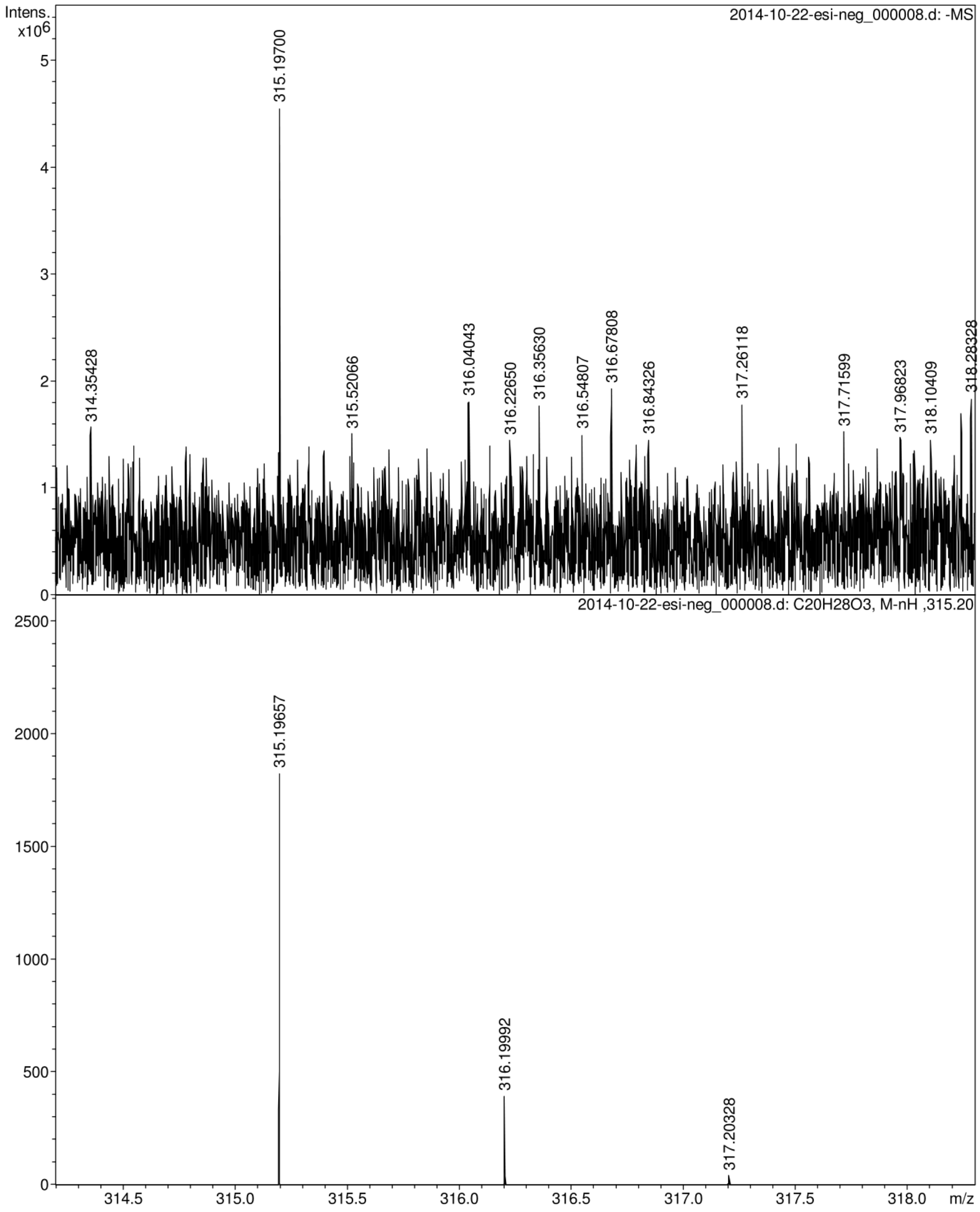

Compound 3

Generic Display Report

Analysis Info

Analysis Name	D:\Data\Nick-2014-files\ESI_Positive\16-October\2014-10-16\2014-10-16-posesi-service_000011.d	Acquisition Date	16/10/2014 11:45:07 AM
Method	1MW Positive ESI	Operator	
Sample Name	Purple	Instrument	apex-Ultra
Comment	MeOH 1 1M TOF delay 0.0006s, Q1 200 m/z		

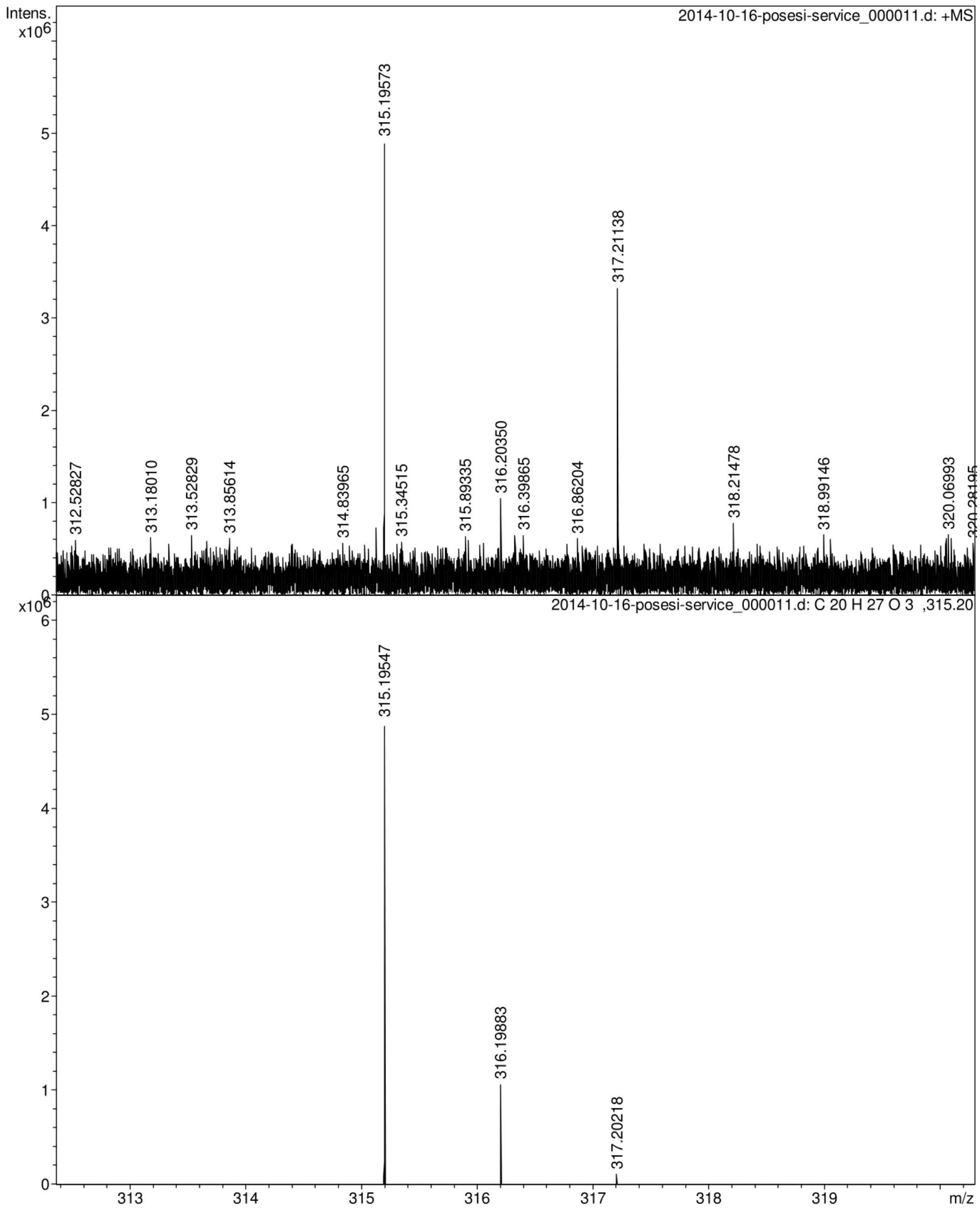

Compound 4

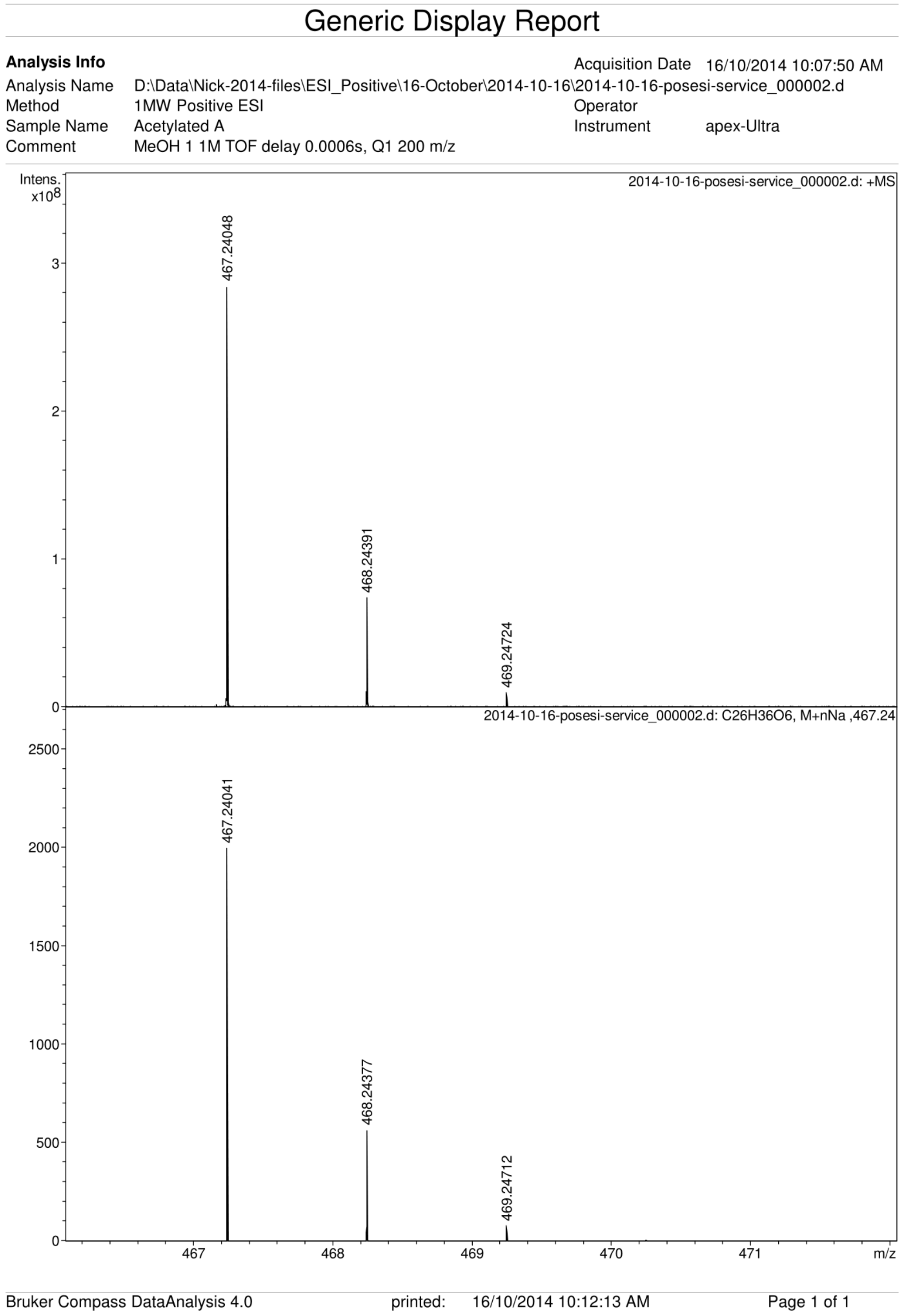
Generic Display Report
Analysis Info
Acquisition Date 16/10/2014 10:07:50 AM
Analysis Name D:\Data\Nick-2014-files\ESI_Positive\16-October\2014-10-16\2014-10-16-posesi-service_000002.d
Method 1MW Positive ESI
Operator
Sample Name Acetylated A
Instrument apex-Ultra
Comment MeOH 1 1M TOF delay 0.0006s, Q1 200 m/z
Intens.
x10^8
2014-10-16-posesi-service_000002.d: +MS
467.24048
468.24391
469.24724
2014-10-16-posesi-service_000002.d: C26H36O6, M+nNa ,467.24
467.24041
468.24377
469.24712
467
468
469
470
471
m/z
Bruker Compass DataAnalysis 4.0
printed: 16/10/2014 10:12:13 AM
Page 1 of 1

Compound 5

Generic Display Report

Analysis Info

Analysis Name	D:\Data\Nick-2014-files\ESI_Positive\16-October\2014-10-16\2014-10-16-posesi-service_000004.d	Acquisition Date	16/10/2014 10:28:02 AM
Method	1MW Positive ESI	Operator	
Sample Name	Acetylated B	Instrument	apex-Ultra
Comment	MeOH 1 1M TOF delay 0.0006s, Q1 200 m/z		

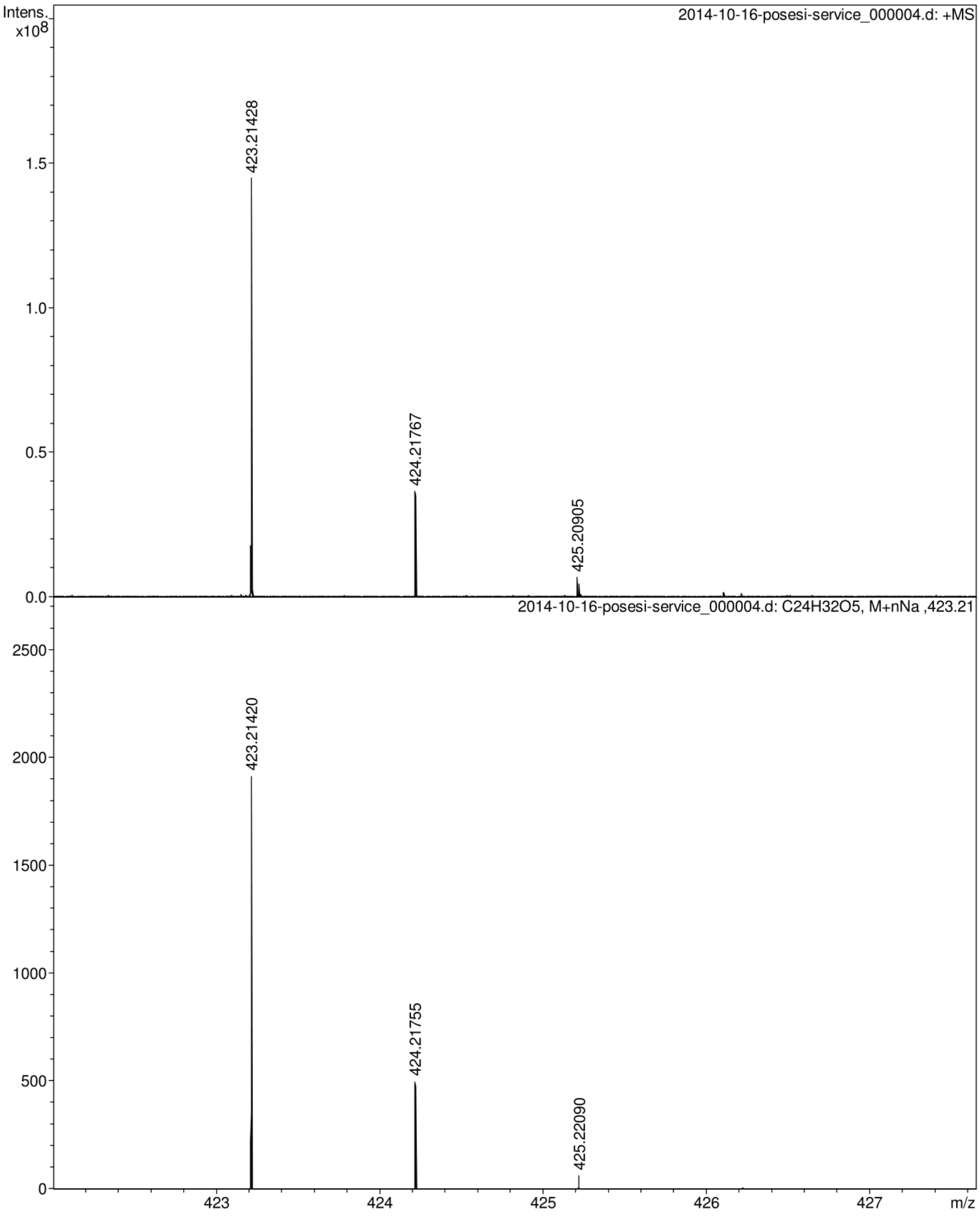

Schematic formation of compound 3

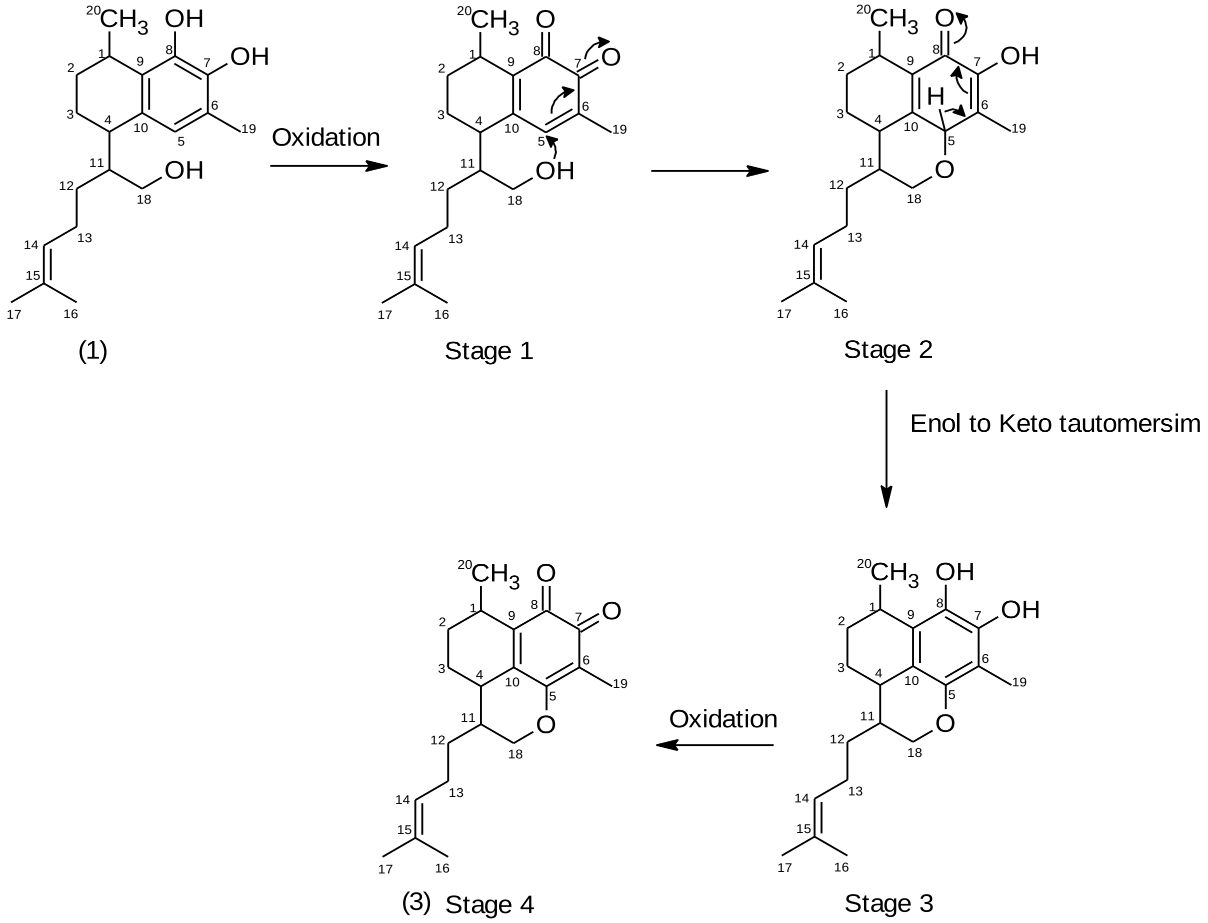

Supplement: Supplementary file 1 [file molecules-22-01185-s001.zip › Appendix A_supporting data.pdf]
